# Bayesian calibration of simulation models for supporting management of the elimination of the macroparasitic disease, Lymphatic Filariasis

**DOI:** 10.1186/s13071-015-1132-7

**Published:** 2015-10-22

**Authors:** Brajendra K. Singh, Edwin Michael

**Affiliations:** Department of Biological Sciences, University of Notre Dame, Notre Dame, IN USA

**Keywords:** Vector-borne neglected tropical diseases, Lymphatic filariasis, Parasite transmission heterogeneity, Uncertainty, Ecological complexity, Transmission models, Infection breakpoints, Indicator dynamics, Data-model assimilation, Bayesian melding, Mass drug administration, Vector control, Extinction dynamics, Decision support, Parasite elimination programmes

## Abstract

**Background:**

Mathematical models of parasite transmission can help integrate a large body of information into a consistent framework, which can then be used for gaining mechanistic insights and making predictions. However, uncertainty, spatial variability and complexity, can hamper the use of such models for decision making in parasite management programs.

**Methods:**

We have adapted a Bayesian melding framework for calibrating simulation models to address the need for robust modelling tools that can effectively support management of lymphatic filariasis (LF) elimination in diverse endemic settings. We applied this methodology to LF infection and vector biting data from sites across the major LF endemic regions in order to quantify model parameters, and generate reliable predictions of infection dynamics along with credible intervals for modelled output variables. We used the locally calibrated models to estimate breakpoint values for various indicators of parasite transmission, and simulate timelines to parasite extinction as a function of local variations in infection dynamics and breakpoints, and effects of various currently applied and proposed LF intervention strategies.

**Results:**

We demonstrate that as a result of parameter constraining by local data, breakpoint values for all the major indicators of LF transmission varied significantly between the sites investigated. Intervention simulations using the fitted models showed that as a result of heterogeneity in local transmission and extinction dynamics, timelines to parasite elimination in response to the current Mass Drug Administration (MDA) and various proposed MDA with vector control strategies also varied significantly between the study sites. Including vector control, however, markedly reduced the duration of interventions required to achieve elimination as well as decreased the risk of recrudescence following stopping of MDA.

**Conclusions:**

We have demonstrated how a Bayesian data-model assimilation framework can enhance the use of transmission models for supporting reliable decision making in the management of LF elimination. Extending this framework for delivering predictions in settings either lacking or with only sparse data to inform the modelling process, however, will require development of procedures to estimate and use spatio-temporal variations in model parameters and inputs directly, and forms the next stage of the work reported here.

**Electronic supplementary material:**

The online version of this article (doi:10.1186/s13071-015-1132-7) contains supplementary material, which is available to authorized users.

## Background

A major requirement for designing and managing effective population-level strategies to interrupt self-sustaining parasite transmission from communities is the reliable quantification of the expected dynamical responses of parasite populations to applied interventions [1–5]. A second key need is the identification of the measurable infection or transmission-related variables that can serve as reliable thresholds for indicating with high certainty that parasite transmission is interrupted or no longer self-sustaining in any given setting [6–9]. Beyond these requirements, two other important needs are the quantification and understanding of how parasite control dynamics and transmission thresholds or breakpoints may: 1) vary due to the uniqueness of a given ecological setting, and 2) are influenced by the extant uncertainty and stochasticity surrounding the system processes that may govern any such local variations in dynamics [10–12]. It is also important to recognize that parasite transmission systems are complex, adaptive and evolvable, whose behaviour emerges from the interaction of adapting components [11, 13]. Their behaviour is thus frequently nonlinear, sometimes managing to resist large perturbations and other times transforming because of small perturbations [7, 13–15]. These considerations imply that the design and management of parasite elimination programs, and indeed assessment of the controllability of a given parasite system, are primarily tied to gaining a better understanding as well as incorporation of the underlying complexity, uncertainty and heterogeneity inherent in the transmission dynamics effectively into management practice [8, 10, 16–18]. The dynamical nature of the problem also highlights that insights from empirical field or clinical trial results alone will not provide the information required to make the programmatic decisions regarding the best strategies to identify thresholds, break transmission and when to stop parasite interventions [8, 19–23].

The vital roles that mathematical models of parasite transmission can play in addressing these questions and hence support the effective design and assessment of community-based intervention programs have long been noted ([1, 24]), and yet the actual use of these process-based tools for guiding parasite elimination has so far been limited [25]. Partly, we indicate that this is linked to the fact that the application of any modelling construct involves substantial variabilities, contributed partially by epistemic uncertainties regarding model structure and parameters, and in part by the often limited quantity and quality of data inputs [10, 18, 26, 27]. This difficulty is further exacerbated when local transmission dynamics vary between communities owning to variations in boundary or initial conditions, which will lead to misleading locality-specific control predictions by models that base their parameters on averaged universal values [28, 29]. A second difficulty is that most models developed to depict the average behaviour of a system are inadequate for addressing the type of percentile- or probabilistic-based outcomes needed by policy makers in order to make decisions that can reliably accommodate the variability that may occur in system response to different interventions applied variously across diverse transmission settings. These challenges suggest that to enhance the credibility and value of using transmission models for guiding decision support, both the uncertainty in model predictions as well as the full range of expected system responses across different local settings must be rigorously quantified if we are to provide the type of dynamics-based policy informatics required to more effectively achieve successful parasite control everywhere [10, 18].

This report is of work in progress in our laboratory aimed at developing a modelling framework that can effectively address each of the above issues, in the context of improving the use of mathematical models of the major vector borne macroparasitic disease, lymphatic filariasis (LF), as tools for guiding the design of management strategies for the many locations where the disease is endemic. In particular, we illustrate and highlight features of a Bayesian methodology that can facilitate the combination of LF transmission models with data in a statistically rigorous way so as to constrain model parameters and system states, identify and quantify model errors, and improve predictions of parasite transmission and control in different ecological settings [10, 18, 26, 30]. Our chief rationale in developing this methodology stems from the belief that to be most useful for predictive epidemiology, we need both process or mechanistic models to represent the key biological structures, components and processes that determine the dynamical behaviour of a parasite transmission system, and also data, to quantify these processes and components, and thereby constrain the resultant model parameters and state variables via data assimilation [31–33]. A feature of such modelling schemes is how they can also incorporate information that pertains to uncertainty of both the model and the observations, and thus permit better estimates of the true state of a dynamical system in a locality [34]. Additionally, as estimates of model output uncertainty are calculated in accordance with the observed data, model predictions of quantities of interest (*eg.* thresholds, infection patterns, timelines to elimination) are also correlated to their associated probabilistic density functions derived from the particular method used to match sets of model simulation outputs to data [26, 28, 34, 35]. This expression of model outputs as a probability distribution is crucial to allowing policy makers to make decisions that accommodate the natural variability of dynamical systems, as well as the uncertainty inherent in model predictions due to incomplete understanding of infection dynamics in any given endemic setting.

The analysis reported here begins with a presentation of the specifics of the Bayesian data calibration framework we have developed for supporting LF modelling based on field data. This is followed by describing results from the model calibration exercise, and examination of the ability of vector-specific LF models to adequately reproduce the infection patterns observed in human communities from across settings representing the major LF endemic regions of the world. We then use these locally calibrated models to address several critical questions regarding the design of LF management strategies for successfully achieving parasite elimination, including: 1) what types of eradication thresholds, and what would the values of these thresholds be, that provide the best means to assess LF elimination and which therefore may adequately serve as elimination targets for LF intervention programs; 2) are the values of these thresholds consistent across different local settings, and what might the implications of any heterogeneity be for the frequency and durations of interventions required to break parasite transmission in any given site; and 3) which of the current LF strategies and what remedial measures may best overcome these heterogeneities in extinction dynamics so that the likelihood of achieving LF elimination may be accomplished within a reasonable time frame across all settings? We end by discussing the importance of our results for the global LF elimination programme, by assessing the probabilities of both achieving infection elimination and reducing risk of infection recrudescence locally using the globally-set World Health Organization (WHO) thresholds and recommended mass treatment regimens and schedules.

## Methods

### Data

The data used in this analysis were compiled from published pre-control cross-sectional surveys of microfilariae (mf) prevalence and mosquito abundance carried out in 22 geographically-distinct communities across the major LF endemic settings of Africa, Southeast Asia and Papua New Guinea in the Pacific region. These datasets were selected on the basis that they provide human age-mf prevalence data, stratified by age-classes of individuals sampled and numbers of mf-positives out of these samples, information on the dominant vector species, and measurements of the corresponding annual biting rates (ABR) of mosquitoes indicating the vector transmission intensity prevailing in each site. Five out of these 22 sites also have age-profile data on circulating filarial antigen (CFA). Details of the data - sample sizes and % mf and CFA positives, along with sampling blood volumes used to assess infection prevalence, dominant vector species and ABRs - for each of the 22 survey sites are given in Table 1. Information on the drug regimen used for simulating the effects of interventions in each of these sites by MDA without/with vector control (VC) are also given, reflecting the current WHO guidelines and use of drug combinations advocated for these sites.

**Table 1:** Description of baseline survey data. The study sites are given with the baseline sample size and microfilariae (mf) prevalence (%), blood volumes collected during the survey to test for mf positivity, annual biting rate (ABR) of vector mosquitoes, dominant vector species and drug regimen used for simulating the chemotherapeutic interventions by mass drug administration (MDA) without/with vector control (VC)

Study villages	Mf Sample size	Blood volume (*μl*)	^c^Mf (%)	^d^Baseline ABR	CFA sample size	CFA (%)	Mosquito species (genus)	^a^Drug regimen	^b^Drug’s efficacies (*ω*, *ε*, T_P_)	Source
Peneng	63	1000	66.67	8194	-	-	*An.*	DEC + ALB	(55, 95, 6)	[10, 18, 78, 79]
Albulum	50	1000	80	42328	-	-	*An.*	DEC + ALB	(55, 95, 6)	[10, 18, 78, 79]
Yauatong	131	1000	92.37	37052	-	-	*An.*	DEC + ALB	(55, 95, 6)	[10, 18, 78, 79]
Nanaha	211	1000	54.98	11611	-	-	*An.*	DEC + ALB	(55, 95, 6)	[10, 18, 78, 79]
Ngahmbule	346	1000	51.16	4346	-	-	*An.*	DEC + ALB	(55, 95, 6)	[10, 18, 78, 79]
Tawalani	367	100	35.72	12850	-	-	*An.*	IVM + ALB	(35, 99, 9)	[80]
^e^Jaribuni	1007	100	25.35	15677	-	-	*An.*	IVM + ALB	(35, 99, 9)	[81, 82]
Tingrela	699	20	63.89	4156	-	-	*An.*	IVM + ALB	(35, 99, 9)	[83]
Chiconi	245	20	58.90	10586	-	-	*An.*	IVM + ALB	(35, 99, 9)	[84]
^e^Masaika	848	100	28.61	6184	837	52.2	*An.*	IVM + ALB	(35, 99, 9)	[85]
^e^Kirare	919	100	28.18	2090	90	53.3	*An.*	IVM + ALB	(35, 99, 9)	[86]
^e^Alebtong	739	100	33.47	58292	890	29.1	*An.*	IVM + ALB	(35, 99, 9)	[87]
^e^Lwala	572	100	21.05	16341	896	18.3	*An.*	IVM + ALB	(35, 99, 9)	[87]
^e^Obalanga	799	100	34.62	4587	900	30.1	*An.*	IVM + ALB	(35, 99, 9)	[87]
^e^Kingwede	825	100	3.07	1548	-	-	*Cx.*	IVM + ALB	(35, 99, 9)	[85]
^e^Mao	546	100	27.8	25439	-	-	*Cx.*	IVM + ALB	(35, 99, 9)	[80]
^e^Mambrui	787	100	24.99	4964	-	-	Cx.	IVM + ALB	(35, 99, 9)	[81, 82]
Pondicherry	1549	20	34.74	88500	-	-	*Cx.*	DEC + ALB	(55, 95, 6)	[88]
Calcutta	861	20	26.72	115942	-	-	*Cx.*	DEC + ALB	(55, 95, 6)	[89, 90]
Vettavallam	7976	20	22.83	100375	-	-	*Cx.*	DEC + ALB	(55, 95, 6)	[91]
Pakistan	1443	20	31.49	1607	-	-	*Cx.*	DEC + ALB	(55, 95, 6)	[92, 93]
Jakarta	922	20	12.27	223000	-	-	*Cx.*	DEC + ALB	(55, 95, 6)	[94]

*An*.: *Anopheles* mosquitoes; *Cx*.: *Culex* mosquitoes; Drug’s efficacies (ω,*ε*, *T*_*P*_): (instantaneous kill rate (%) for adult worms, instantaneous kill rate (%) for mf, drug’s efficacy period in months); mf (%): mf prevalence in percentages calculated from the number of mf-positive samples out of the total individuals sampled (sample size) in a study site; DEC: diethylcarbamazine citrate; IVM: Ivermectin; ALB: Albendazole.

^a^The combination drug regimens used for MDA simulations in each site areas are as recommended for each site region by the WHO [95, 96].

^b^Drug efficacy values are taken from [3].

^c^All mf prevalence values were standardized to reflect sampling of 1 ml blood volumes using a transformation factor of 1.95 and 1.15 respectively for values originally estimated using 20 or 100 *μl* blood volumes [49].

^d^Baseline ABR are used to obtain the monthly biting rate (MBR = ABR/12), which is used as inputs into the LF models described in the text. The symbol (−) indicates that the baseline CFA data are either not available or available but not broken by age-groups.

^e^These sites have mixed vector species: here they are represented by the dominant vector species.

### The mathematical model of LF transmission dynamics

We extended the recently developed mosquito genus-specific LF transmission model [3, 7, 10, 18, 22] to carry out the modeling work in this study by including two new state variables into the model. The first variable is included to capture the effect of pre-patency (time interval between infection establishment and the age at which worms become sexually mature and female worms begin to produce microfilariae) in mf production, which in LF is thought to generally last for a period of 6–9 months [36]. Such lengthy pre-patent periods can introduce a significant lag into the worm dynamics, including influencing system breakpoints and responses to perturbations [37]. The second state variable is included to capture and investigate the dynamics of a key proposed indicator of LF infection in a population, *viz.* Circulating Filarial Antigen (CFA), which is thought to be a better marker of infection than mf particularly when parasite populations are reduced to low levels by repeated interventions. As in the previous version, the state variables of this extended system vary over age (*a*) and/or time (*t*), representing changes in the pre-patent and patent worm burden per human host, respectively denoted by *P*(*a*, *t*) and *W*(*a*, *t*); the mf level in the human host modified to reflect infection detection in a 1 ml blood sample (*M*(*a*, *t*)); the average number of infective L3 larval stages per mosquito (*L*); a measure of immunity (*I*(*a*, *t*)) developed by human hosts against L3 larvae; and intensity of CFA (denoted by *A*(*a*, *t*)). These states of the model are represented by the following coupled system of partial and ordinary differential equations:

$$\begin{array}{l}\frac{\partial P\left(a,t\right)}{\partial t}+\frac{\partial P\left(a,t\right)}{\partial a}=\lambda \frac{V}{H}h(a)\varOmega \left(a,t\right)-\mu P\left(a,t\right)-\lambda \frac{V}{H}h(a)\varOmega \left(a,t-\tau \right)\xi \\ {}\frac{\partial W\left(a,t\right)}{\partial t}+\frac{\partial W\left(a,t\right)}{\partial a}=\lambda \frac{V}{H}h(a)\varOmega \left(a,t-\tau \right)\xi -\mu W\left(a,t\right)\\ {}\frac{\partial M\left(a,t\right)}{\partial t}+\frac{\partial M\left(a,t\right)}{\partial a}=\alpha s\phi \left[W\left(a,t\right),k\right]W\left(a,t\right)-\gamma M\left(a,t\right)\\ {}\frac{\partial I\left(a,t\right)}{\partial t}+\frac{\partial I\left(a,t\right)}{\partial a}={W}_T\left(a,t\right)-\delta I\left(a,t\right)\\ {}\frac{\partial A\left(a,t\right)}{\partial t}+\frac{\partial A\left(a,t\right)}{\partial a}={\alpha}_2W\left(a,t\right)-{\gamma}_2A\left(a,t\right)\\ {}\frac{dL}{dt}=\lambda \kappa g{\displaystyle \int \pi (a)\Big(1-f\left[M\left(a,t\right)\right]}\Big)da-\sigma L-\lambda {\psi}_1L\\ {}{L}^{*}=\frac{\lambda \kappa g{\displaystyle \int \pi (a)\Big(1-f\left[M\left(a,t\right)\right]}\Big)da}{\sigma +\lambda {\psi}_1}.\end{array}$$

The quantity L^*^ denotes the equilibrium density of infective L3 larvae, and *Ω*(*a*, *t*) = L^*^ψ_1_*ψ*_2_*g*_1_[*I*(*a*, *t*)]*g*_2_[*W*_*T*_(*a*, *t*)] is the establishment rate of larvae in the human host moderated by the effects of acquired immunity (as modelled by function *g*_1_[*I*(*a*, *t*)]) and/or immune tolerance (modelled by function *g*_2_[*W*_*T*_(*a*, *t*)]) [10, 18]. The above equations involve partial derivatives of five state variables (*P* and *W* - pre-patent and adult worm loads; *M* - microfilaria intensity; *I* - immunity to acquiring new infection due to the pre-existing total worm load (*W*_*T*_ = *P* + *W*); and *A* - intensity of circulating filarial antigen (CFA)), whereas given the faster time scale of infection dynamics in the vector compared to the human host, the infective L3-stage larval density in the mosquito population is modeled by an ordinary differential equation, essentially reflecting the significantly faster time-scale of larval infection dynamics in the vector hosts. This allows making the simplifying assumption that the density of infective stage larvae in the vector population reaches a dynamic equilibrium (denoted by L^*^) rapidly [7, 10, 18, 38, 39]. Note that we capture the effects of worm patency by considering that at any given time *t*, human individuals of age less than or equal to pre-patency period, *τ*, will have no adult worms or microfilariae, *ie.*$W\left(a,t\right)=M\left(a,t\right)=\mathsf{0}$ for *a* ≤ *τ*, and the rate at which pre-patent worms survive to become adult worms in these individuals at *a* > *τ* is given by *ζ* = exp(−*μτ*). The term *f*[*M*(*a*, *t*)] describes the functional form relating the L3-stage larval uptake and development in the vector population, which is known to differ significantly in the two major genus of mosquito vectors implicated in LF transmission [40–43]. The derivation of these two larval uptake and development functions are given elsewhere [7]. This basic immigration-death model structure as well as recent extensions have been discussed [7, 10, 18, 38, 39]; see Additional file 1: Table S1 for the description of all the model parameters and functions.

### The Bayesian melding method

#### Notation

Following [28], we begin by denoting the collection of model inputs about which information is uncertain by *Θ*. These can include model parameters and starting values of a system. We note that this collection of model inputs may represent a subset of the set of model inputs, and does not consist of those inputs that, based on expert knowledge and experience, are taken to be known or fixed in the model. We represent the collection of model outputs about which we have observed information (such as the number of mf positives from the baseline LF surveys) by *Φ*. This collection will be a subset of all the model outputs, and can include values of a few or all state variables of the system of interest at various time-points. Note, in the case of a deterministic system, we can derive a mapping function by *M*_*Φ*_ such that *Φ* = *M*_*Φ*_(*Θ*), *ie.* the outputs are fully expressed in terms of the inputs. The third notation represents the quantities of policy or research interest, and is denoted by *Ψ*. These quantities of interest can be functions of either model inputs or outputs, or of both, such that: *Ψ* = *M*_*Ψ*_(*Θ*, *Φ*) = *M*_*Ψ*_(*Θ*, *M*_*Φ*_(*Θ*)), which shows that *Ψ* can be represented as a function of the inputs alone. Finally, data collected from affected populations/communities provide information about the model outputs. Collection of such data is represented by *y*.

#### Bayesian melding

The basic idea under this method is to combine or fuse all available information about model inputs and model outputs via Bayesian synthesis, in order to yield a Bayesian posterior distribution of the quantities of interest, *Ψ*. The first step under the BM method is thus to translate and encode the available information about model inputs and outputs in terms of probability distributions. This can be done as follows. We represent the available information about the inputs, *Θ*, by a prior probability distribution *q*(*Θ*). We specify a conditional probability distribution of the data *y* given the outputs *Φ*, and this yields a likelihood for the outputs, which can be represented as *L*(*Φ*) = Prob(*y*|*Φ*). As an aside, in the case of the modelling of LF infection age-profiles, this likelihood can be evaluated using a binomial probability function:

$$\Pr \left(Y=y\right)=\frac{n!}{y!\left(n-y\right)!}{p}^y{\left(1-p\right)}^{n-y}$$

where *y* is the number of mf-positive blood samples out of the total *n* blood samples collected during the baseline survey conducted in a LF endemic site with *p* being the probability of such observation in different age-classes. In order to obtain a joint fit of the model to parallel CFA and mf age-data where both are available, this binomial probability function was modified and used following the proposal made in [44] by:

$$\Pr \left({Y}_1={y}_1,{Y}_2={y}_2\right)=\frac{n_1!}{y_1!\left({n}_1-{y}_1\right)!}{p_1}^{y_1}{\left(1-{p}_1\right)}^{n_1-{y}_1}\times f\left({y}_2\Big|{y}_1\right),$$

where

$$\begin{array}{c}f\left({y}_2\Big|{y}_1\right)={\left(1+{\alpha}_{mix}\right)}^{-{n}_1}{\displaystyle \sum_{\left({j}_1,{j}_2,{j}_3\right)\in S}\frac{y_1!}{j_1!\left({y}_1-{j}_1\right)!}\frac{\left({n}_1-{y}_1\right)!}{j_2!\left(\left({n}_1-{y}_1\right)-{j}_2\right)!}}\frac{\left({n}_2-{n}_1\right)!}{j_3!\left(\left({n}_2-{n}_1\right)-{j}_3\right)!}{\left\{{p}_2+{\alpha}_{mix}\left({p}_2-{p}_1\right)+{\alpha}_{mix}\right\}}^{j_1}\\ {}\\ {}\times {\left\{1-{p}_2+{\alpha}_{mix}\left({p}_2-{p}_1\right)\right\}}^{\left({y}_1-{j}_1\right)}{\left\{{p}_2+{\alpha}_{mix}\left({p}_2-{p}_1\right)\right\}}^{j_2}\\ {}\\ {}\times {\left\{1-{p}_2+{\alpha}_{mix}\left({p}_2-{p}_1\right)+{\alpha}_{mix}\right\}}^{\left({n}_1-{y}_1-{j}_1\right)}{p_2}^{j_3}{\left(1-{p}_2\right)}^{\left({n}_2-{n}_1-{j}_3\right)},\\ {}\end{array}$$

with *S* = {(*j*_1_, *j*_2_, *j*_3_) : *j*_1_ + *j*_2_ + *j*_3_ = *y*_2_; *j*_1_ = 0, 1, …, *y*_1_; *j*_2_ = 0, 1, …, *n*_1_ − *y*_1_; *j*_3_ = 0, 1, …, *n*_2_ − *n*_1_}.

In the above, *y*_2_ and *y*_1_ are represented by CFA and mf positives in different age-classes, with *n*_2_ ≥ *n*_1_ respectively representing the CFA and mf samples collected during the survey. The term *α*_*mix*_ is a mixing parameter. As per the proposal in [44], the alternate case, *n*_1_ ≥ *n*_2_, can also be similarly derived. The use of this joint likelihood function for fitting the LF model simultaneously to these two variables (*ie*. CFA and mf) is justified as these are both functions of the underlying adult worm burdens (see the LF system equations) and thus are correlated.

As for outputs (see above), a conditional likelihood for the inputs is expressed as follows: *L*(*Θ*) = Prob(*y*|*M*_*Φ*_(*Θ*)). As we have both a prior probability density function (*q*(*Θ*)) and a likelihood function (*L*(*Θ*)) for the inputs, following Bayes’s theorem we can obtain a posterior distribution of the inputs given all the available information. This posterior distribution density is proportional to the prior density times the likelihood of the inputs given data, and can be expressed as:

$$\pi \left(\varTheta \right)\propto q\left(\varTheta \right)L\left(\varTheta \right).$$

A constant of proportionality can be defined given this expression such that *π*(*Θ*) becomes a probability density. In other words, it integrates to 1 over the joint space of the inputs with a suitable choice of proportionality constant. As the quantities of policy/research interest (*Ψ*) can be expressed in terms of the inputs, the posterior probability distribution of the inputs yields a posterior distribution of the quantities of interest, which is denoted as *π*(*Ψ*). This posterior distribution *π*(*Ψ*) thus combines all the available information on the inputs and outputs of a system in a statistically coherent way, and therefore may provide a comprehensive basis for carrying out risk assessments and decision-making about a dynamical entity [29].

#### Simulating the posterior distribution

It is clear that for a complex model and due to various mapping functions, namely *M*_*Φ*_ and *M*_*Ψ*_, the posterior distribution *π*(*Θ*) will not have an analytic form. However, since these mapping functions can be evaluated via computer simulations, the use of a Monte Carlo method based on the sampling importance resampling (SIR) algorithm can approximate this posterior distribution [10, 18, 29, 35]. This works as follows:

1. Draw a sample {*Θ*_1_, , *Θ*_*I*_} of values of the inputs from the prior distribution *q*(*Θ*) with *I* = 200000. Note that, each element (referred to as a parameter vector in this paper) of this collection comprises of all model parameters, and, in practice, the value of *I* can vary between 50,000 and 200,000 [10, 18, 45]. In other words, this sample of *Θ* has a set of *I* parameter vectors. The random values of the inputs can be drawn for the appropriate distributions. In our case the inputs are drawn from the uniform distribution with the extremes set by the known minimum and maximum values of the model parameters based on expert knowledge and experience. See Additional file 1: Table S1 for the maximum and minimum of the model parameters.
2. Obtain the collection of the corresponding model outputs {*Φ*_1_, , *Φ*_*I*_} with the mapping function of *Φ*_*i*_ = *M*(*Θ*_*i*_) as defined above. In other words, the collection of the outputs is generated by simulating the dynamic model for all elements of the input collection {*Θ*_1_, , *Θ*_*I*_}.
3. Compute weights for each of the elements in the collection of the outputs given data using *w*_*i*_ = *L*(*y*|*Φ*_*i*_). Employing the mapping function which relates the outputs with the inputs, we thus get the weights (*ie.*, *w*_*i*_ = *L*(*y*|*M*_*Φ*_(*Θ*_*i*_))) for all *Θ*_*i*_.
4. Use the SIR algorithm to approximate the posterior distribution of the inputs with values {*Θ*_1_, , *Θ*_*I*_} by resampling them (at least, a set of *l* = 500 [10, 18, 45]) from the collection with probabilities proportional to {*w*_1_, *w*_*I*_}.
5. Use the posterior distribution of the inputs to approximate the posterior distribution of the quantities of interest. The approximated posterior distribution has values {*Ψ*_1_, , *Ψ*_*I*_} where *Ψ*_*i*_ = *M*_*Ψ*_(*Θ*_*i*_, *Φ*_*i*_) and probabilities proportional to {*w*_1_, ⋯, *w*_*I*_}. In practice, the posterior distribution of the quantities of interest is obtained by re-running the dynamic model of the system under investigation over the resampled set (*cf.* Step 4) of the inputs. For example, we obtain the model fits to the observed mf age-profile data by re-running the model using the posterior distribution of the inputs. Similarly, the posterior is used to calculate the infection breakpoints and/or threshold biting rates (TBRs), which are then used to calculate the timelines of LF elimination under a set of intervention scenarios as discussed below.

### Numerical stability analysis for quantifying infection breakpoints and vector biting thresholds

A previously developed numerical stability analysis procedure, based on varying initial values of L^*^ to each of the SIR selected model parameter sets or vectors, was used to calculate the distribution of mf, and for the first time, CFA, prevalence breakpoints and the corresponding threshold biting rates (TBR) that may be expected in each study community [10, 18], as follows. Briefly, we begin by progressively decreasing *V/H* from its original value to a threshold value *below* which the model always converges to zero mf prevalence, regardless of the values of the endemic infective larval density L^*^. The product of *λ* and this newly found *V/H* value is termed as the threshold biting rate (TBR). Once the threshold biting rate is discovered, the model at TBR will settle to either a zero (trivial attractor) or non-zero mf prevalence depending on the starting value of L^*^. Therefore, in the next step, while keeping all the model parameters unchanged, including the new V/H and by starting with a very low value of L^*^ and progressively increasing it in very small step-sizes we estimate the minimum L^**^below which the model predicts zero mf prevalence and above which the system progresses to a positive endemic infection state. Here, thus, L^**^ represents the L3 breakpoint density in the vector population. This value can be converted to a prevalence value using the relationship: *P*(*Z*, *k*) = 1 − (1 + *Z*/*k*)^− *k*^, where *P* is the L3 infection prevalence, *Z* is the L3 density (ie., L^**^) and *k* is the aggregation parameter of the negative binomial distribution [46]. The mf prevalence at the L^**^ value is termed as the worm/mf breakpoint in this study [7]. Similarly, given the coupling to CFA predictions, we may also evaluate the corresponding CFA prevalence at L^**^, which is termed as the CFA breakpoint. The collections of mf and CFA breakpoints, and the L3 breakpoint prevalences, from the SIR selected parameter vectors in a site, are then used to get the LF infection extinction thresholds signifying various probabilities of elimination following the method outlined in [47]. Note, however, that here we focus on the 95 % elimination probability threshold to serve as targets for the intervention simulations described below.

### Modeling intervention by mass drug administration

Intervention by MDA was modeled based on the assumption that anti-filarial treatment with a combination drug regimen acts, firstly, by killing certain fractions of the populations of adult worms and mf instantly following drug administration. These effects are incorporated into the basic model by calculating the drug-induced removal of worms and mf:

$$\left.\begin{array}{l}W\left(a,t+dt\right)=\left(1-\omega C\right)W\left(a,t\right)\\ {}M\left(a,t+dt\right)=\left(1-\varepsilon C\right)M\left(a,t\right)\end{array}\right\}\kern1.25em \mathrm{at}\ \mathrm{time}\ t={T}_{MD{A}_i}$$

where *dt* is a short time period since the time point ${T}_{MD{A}_i}$ when the *i*^th^ MDA was administered. The parameters *ω* and *ε*, are drug killing efficacy rates for the two life stages of the parasite while the parameter *C* represents the drug coverage. Apart from instantaneous killing of mf, the drug is also thought to continue to kill the newly reproduced mf by any surviving female adult worms for a period of time *T*_*p*_. We model this effect as follows:

$$\frac{\partial M\left(a,t\right)}{\partial t}+\frac{\partial M\left(a,t\right)}{\partial a}=\left(1-\varepsilon C\right)\alpha s\phi \left(W\left(a,t\right),k\right)W\left(a,t\right)-\gamma M\left(a,t\right)\kern0.5em ,\kern1em \mathrm{f}\mathrm{o}\mathrm{r}\ {T}_{MD{A}_i}<t\le {T}_{MD{A}_i}+{T}_p.$$

#### Simulating LF MDA interventions

We simulated the effects of MDA interventions by running the model with fixed-values of the three drug-related parameters (*ω*, *ε* and *T*_*p*_) for MDA coverage levels ranging from 60 to 100 %. The values of worm and mf kill rates for the drug regimens studied here, *viz.* DEC+ ALB and IVM + ALB (Table 1) were taken from [3]. The first MDA round is implemented in the model by applying the above equations to the parameter vectors obtained from the baseline fits describing the pre-control worm (*W*) and mf (*M*) loads in each site, and subsequent interventions are simulated as periodic (yearly and 6-monthly, respectively, for annual and biennial MDAs) events acting on parasite loads resulting from each sequentially applied MDA. We investigated the impact of annual and biennial MDAs on the years of mass treatment required to reduce mf prevalence from baseline to below the elimination threshold estimated for each site. In the model, the effect of drugs was modelled in the subpopulation of 5 years old and above.

### Modeling vector control by LLINs

We modelled supplemental vector control (VC) applications in terms of the impact of long lasting insecticidal nets (LLINs) by assuming that population-level coverage of LLINs would reduce the vector biting rate to the same degree regardless of the mosquito genus present in a study site. Insecticides used in LLINs have three important effects on vector mosquitoes [4, 48]: they deter mosquitoes from entering human dwellings; inhibit them from taking human blood meals; and kill them. These three effects can be combined for modelling the effect of VC applications on the prevailing ABR. Note that the VC efficacies decay over time, for example, due to wear and tear of bed nets used in the households [4, 48]. We also assume that the LLINs applications in households are regularly replenished or renewed over a recommended time-period [48]. In this study, we consider this period to be of three years for LLINs. Taking into account the decay and periodic replenishment of insecticides, the impact of VC in this work is modelled by extending our previous formulation [3, 39]; whereby we replace $\frac{V}{H}$ in the model equation by the term: $\frac{V}{H}\left(1-{\eta}_1 \exp \left[-\varLambda t\right]{C}_{VC}\right)\left(1-{\eta}_2 \exp \left[-\varLambda t\right]{C}_{VC}\right)\left(1-{\eta}_3 \exp \left[-\varLambda t\right]{C}_{VC}\right)$, where *C*_*VC*_ is the mean annual coverage level in terms of the fraction/percentage of households using LLINs in a LF endemic setting. The parameters ${\eta}_{\mathsf{1}},{\eta}_{\mathsf{2}},{\eta}_{\mathsf{3}},$ respectively, denote the level of deterrence, feeding inhibition and toxicity of the insecticides used in the manufacturing of LLINs, whereas *Λ* is the efficacy decay rate of the insecticides. The efficacy values of these parameters vary depending on the insecticides used in LLINs [4, 48]; see Additional file Table S2 in [48]. In this study, the values of three efficacy parameters (*ie., η*_1_, *η*_2_, *η*_3_) used are (0.2, 0.9, 0.95) for LLINs, which were obtained by averaging across the set of insecticides used in the manufacturing of LLINs for which we have the data for the three efficacy variables. The decay rate (*Λ*) was fixed at 0.26/year which yields the average half-life of the chemicals used in LLIN insecticides of about 3 years (*ie.* LLINs remain, at least, 50 % efficacious before they are replenished with new ones). In this study, all VC results are presented for the household LLIN coverage of 80 %.

### Calculating extinction and recrudescence probabilities

We calculated the probability that LF extinction has been achieved in each study site using the WHO-recommended threshold of 1 % mf by first deriving the empirical inverse cumulative density functions (ICDFs) for the ensemble of mf breakpoint prevalence values obtained in each site. The site-specific probability of LF extinction was then estimated by calculating the exceedance probability of crossing below the 1 % threshold given the derived ICDFs in each of the present sites [47]. These calculations were carried out using the model-estimated mf breakpoint values at both the prevailing ABRs and TBRs in each site. The recrudescence probability for a given study site was calculated simply as the percentage of the total SIR selected model runs in which the community-level mf prevalence after treatment had been stopped managed to revive and sustain itself above the WHO threshold of 1 % mf prevalence by the end of the period of intervention simulation.

## Results

### Model fits to baseline mf age-prevalence data

**Fig. 1:**
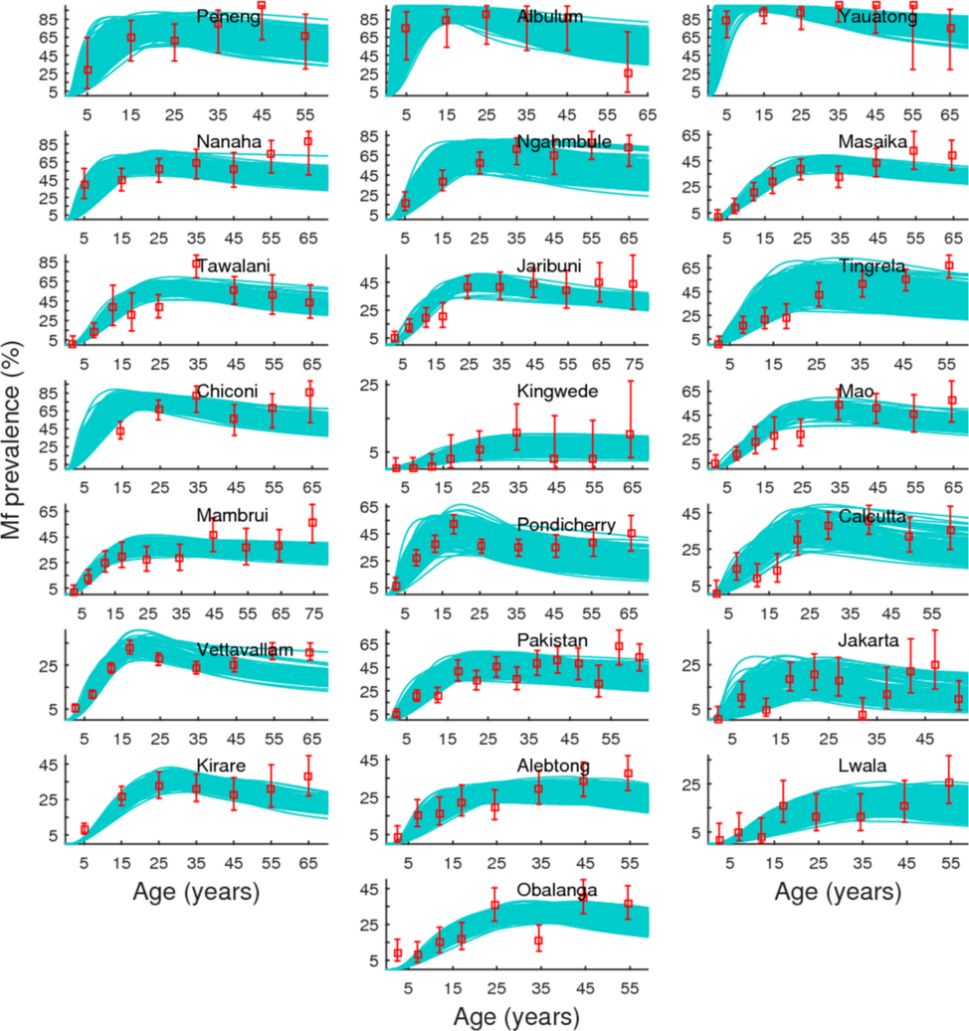
Observed and fitted microfilariae (mf) age-prevalences of lymphatic filariasis (LF) for twenty-two study sites. The cyan *lines* denote the SIR BM model fits to observed baseline mf prevalences in different age-groups (red *squares* with binomial error-bars) from each of the 22 study sites. The age-groups in the figures are represented by the mid-point of the groups studied in each community. Study sites and details of survey data are described in Table 1. All mf prevalence values were standardized to reflect sampling of 1 ml blood volumes using a transformation factor of 1.95 and 1.15 respectively for values originally estimated using 20 or 100 *μL* blood volumes [49]

The 500 SIR generated fits by the genus-specific LF models (*cyan* curves) to the respective baseline mf prevalences in different age-groups (*red* squares representing the means with 95 % binomial confidence intervals) from each of the 22 study sites are shown in Fig. 1. All mf prevalence values were standardized to reflect sampling of 1 ml blood volumes using a transformation factor of 1.95 and 1.15 respectively for values originally estimated using 20 or 100 *μL* blood volumes [49]. The results show that the BM-based data-model assimilation procedure is able to reproduce the age-stratified mf prevalences consistent with observed data in each of the study communities (overall (*ie.* goodness of fit of the model to data across all age-classes) Monte Carlo *p*- values >0.9 in each case), although the fits to the mf baseline data are comparatively better for the study sites that have the least amount of variability in mf prevalence across all age-cohorts; for example, the modelled age-profiles for the study site Jakarta have a comparatively wider spread partly reflecting the higher variability observed in the data from this site (Fig. 1).

### Posterior distributions of model parameters

**Fig. 2:**
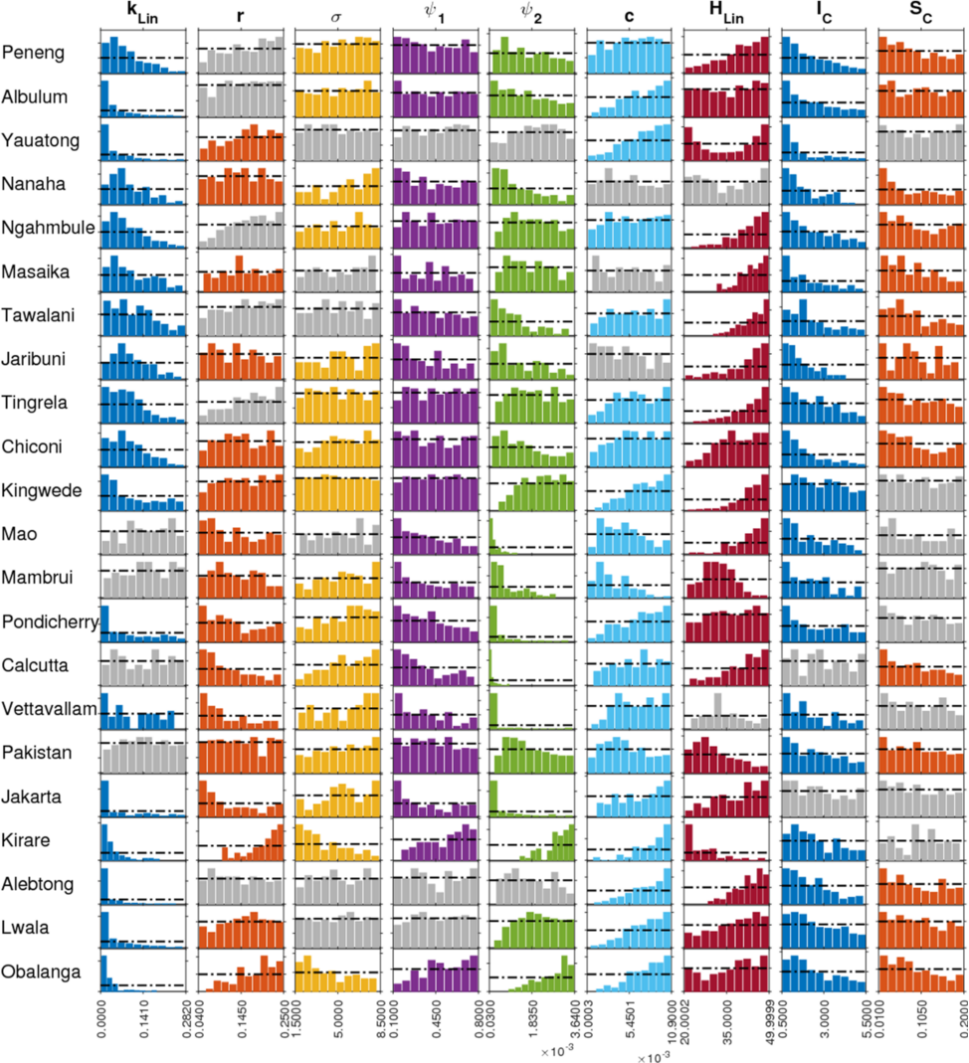
Prior and posterior model parameter distributions for the data from each site. Results are shown for nine parameters (labels on top of each column), which were identified by a formal Kolmogorov-Smirnov (KS) two-sample test, to be significantly different from their flat priors across the majority of the study sites. Distribution plots for a parameter shown in gray denotes that the parameter did not differ significantly from the assigned flat priors for a study site

Figure 2 shows the marginal posterior distributions of those model parameters (9 out of the total 20 parameters) which significantly differed from their initially assigned non-informative or flat priors across the majority of the 22 study sites (identified based on assessing differences between the values of prior and posterior distributions of each parameter using a Kolmogorov-Smirnov (KS) two-sample test). Among these nine parameters, the exposure/worm establishment parameters (*ψ*_1_, *ψ*_2_, *H*_*Lin*_) and immunity-related parameters (*c*, *I*_*C*_) were consistently found to be changed from their initial uniform priors for 19 out of the 22 study sites, indicating both that substantial amount of knowledge was gained regarding these parameters after the Bayesian updating of the respective LF models with data, and that local variations in these parameters might constitute the key factor governing the heterogeneity in the LF mf age-prevalences observed between the present study sites. The parameters *I*_*C*_ and *k*_*Lin*_ stand out in terms of the updated model preference for smaller values in the ranges assigned to them initially, implying that immunosuppression may play only a small role in LF transmission dynamics, and that infection aggregation was highly skewed across all these study sites respectively (Fig. 2). All other updated parameters varied unevenly between the study sites, and thus may represent the major transmission variables that constrained LF infection locally in these sites.

### Mf, L3 and TBR extinction threshold values

As described in Methods, we used the SIR-selected parameter vectors in conjunction with a numerical stability analysis procedure to calculate the values of these thresholds in each of our study sites [7]. Given this ensemble nature of our models, we obtained a range of breakpoints for each of these variables, rather than a single point estimate, in each study site, implying that the probability of LF elimination or extinction will vary across the values of such threshold distributions. Here, we used the complementary or inverse cumulative density function (ICDF) of the estimated threshold values, in conjunction with exceedance calculations [47], to derive the mf and L3 breakpoint threshold values (both in terms of community-level prevalences) signifying a 95 % probability of LF elimination, for use as extinction targets for all the intervention simulations carried out in this study. Table 2 provides the actual numerical values estimated for all the 3 thresholds (*ie.* the TBR and mf/L3 infection breakpoints) for all 22 sites. Note that Mf breakpoints and the L3 breakpoints in mosquitoes were estimated at both the prevailing ABR as well as at the model-derived TBRs in each community; these breakpoint values (including the estimated TBRs) varied significantly between the present study sites irrespective of the transmitting vector (*p*-values < 0.0001 in either of the *Anopheles* or *Culex* settings, Table 2).

**Table 2:** Model-estimated mf and L3 breakpoint values for achieving the successful interruption of LF transmission in each of the study sites investigated. Breakpoints are listed in terms of mf and L3 prevalences (%) at 95 % probability of elimination for two situations: 1) at the prevailing vector biting rates (*ie.*, at the observed ABRs) and 2) at the threshold biting rate (TBR) at or below which LF transmission process cannot sustain itself regardless of the level of the infection in human hosts (see text). The first set of the threshold values (at study-specific ABR) is used in modeling the impact of mass drug administration (MDA) alone, while the 2nd set (*ie*., mf breakpoint values estimated at TBR) is applied for modeling the impact when MDA is supplemented by vector control (VC)

Study villages	Mean TBR	Mf bpts at ABR	L3 bpts at ABR	Mf bpts at TBR	L3 bpts at TBR
Peneng	5635	0.035429	0.001116	0.435501	0.021181
Albulum	11025	0.004885	0.000028	0.094346	0.010902
Yauatong	6185	0.002985	0.000021	0.066789	0.008942
Nanaha	9309	0.066568	0.001827	0.919664	0.022993
Ngahmbule	3365	0.058476	0.001996	0.45293	0.02599
Tawalani	10503	0.085207	0.001871	1.07946	0.02192
Jaribuni	13451	0.077864	0.002772	1.112716	0.022068
Tingrela	3310	0.042786	0.001659	0.559656	0.02453
Chiconi	7315	0.033768	0.000781	0.677308	0.018855
Masaika	5390	0.053393	0.002915	0.451975	0.451975
Kirare	1016	0.018212	0.000805	0.721577	0.061739
Alebtong	18703	0.007344	0.000088	0.22969	0.014427
Lwala	6201	0.015075	0.000217	0.259451	0.021331
Obalanga	1600	0.010066	0.000349	0.907932	0.047025
Kingwede	1363	0.022236	0.0021	0.089295	0.014983
Mao	20062	0.019268	0.002728	0.384838	0.038674
Mambrui	4445	0.075622	0.007421	0.885393	0.065253
Pondicherry	40223	0.000476	0.000025	0.041653	0.007822
Calcutta	86719	0.017295	0.001197	0.178704	0.019703
Vettavallam	66478	0.002706	0.000147	0.110904	0.017511
Pakistan	1311	0.034034	0.00316	0.659793	0.047162
Jakarta	88494	0.000171	0.000015	0.028576	0.00373
^a^ *P*-value (*An*)	<0.0001	<0.0001	<0.0001	<0.0001	<0.0001
^a^ *P*-value (*Cx*)	<0.0001	<0.0001	<0.0001	<0.0001	<0.0001

^a^ *P*-values are from results of Kruskal-Wallis rank sum tests for assessing differences in TBRs, L3 and mf breakpoints among sites within each of the Anopheline (*An*) and Culicine (*Cx*) LF settings

### Joint modelling of CFA and mf age-prevalence data

**Fig. 3:**
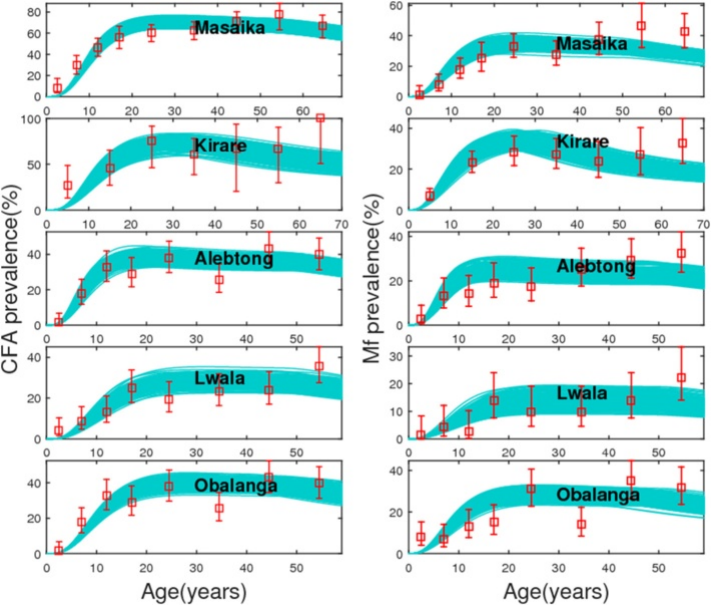
Observed and fitted circulating filarial antigen (CFA) and microfilariae (mf) age-prevalences of lymphatic filariasis (LF) for five study sites. The model fits (cyan *lines*) to the observed baseline CFA and mf age-data (red *squares* with binomial error-bars) were obtained using a bivariate binomial likelihood function as derived and discussed in [44]. Note that both CFA and mf data were available only for five study sites

Figure 3 presents our model fits to the joint baseline CFA and mf age-profile data that were available for analysis from 5 out of 22 sites (Table 1). The resulting estimates of the production and decay/clearance rates of CFA in each community from these joint model fits are displayed in Table 3. These results show that the average production rate (in units of *per worm per month*) ranged from 3.8 to 7, while the CFA clearance rate (in units of *per month*) varied from 0.015 to 0.045 across the 5 study sites. The average survival period of CFA in these communities thus ranged from a little less than two years in 3 of the study sites but to over 3–5 years in the other 2 (see the survival period estimates in Table 3). Apart from signalling the likely longevity of CFA, these results thus indicate intriguingly that a significant clustering of these rates also occurred between the 5 sites. Table 4 presents the joint estimates of the mean TBRs, the threshold values of CFA breakpoints at the community-level as well as in 6 to 7 years old cohort, and values of mf breakpoint, in the 5 study sites. Comparison with the TBR and mf breakpoint values obtained by the single binomial likelihood fits to the mf data for these sites as given in Table 2, indicates that the breakpoint estimates for all 3 thresholds from the joint model fits were distinctly smaller in values (up to in general 3 times for infection thresholds and by at least an order of magnitude for TBR). Note that the estimated CFA breakpoint values are also markedly lower (by order of magnitude of nearly 2) than the WHO recommended values of using either 1 to 2 % in the overall population or <0.1 % in children between 6 and 7 years for assessing disruption of LF transmission in treated communities. These findings may be the outcome of model fitting to parallel infection indicator data, *ie.* to more than one outcome variable, which could lead to a better constraining or estimation of model parameters and hence more reliable predictions of variables of interest. While plausible, it is clear that future work with more data sets providing information on parallel infection measures than currently obtainable is needed to fully evaluate the magnitude of the error that is generated by modelling only mf data versus analysis using simultaneous/joint model fitting to both CFA and mf, and indeed other, indicator data.

**Table 3:** The production and decay/clearance rate parameters for the Circulating Filarial Antigen (CFA) of LF

Study villages	Average	Median	2.5th	97.5th
Production rate				
Masaika	5.69	6.61	4.47	6.61
Kirare	6.49	6.57	4.89	6.66
Alebtong	3.81	3.85	2.51	4.61
Lwala	5.50	5.73	2.97	6.77
Obalanga	7.02	7.09	6.84	7.16
Decay rate				
Masaika	0.028	0.029	0.027	0.029
Kirare	0.015	0.015	0.015	0.024
Alebtong	0.045	0.045	0.037	0.05
Lwala	0.043	0.044	0.031	0.047
Obalanga	0.042	0.042	0.032	0.049
Survival period				
Masaika	35.48	34.78	34.78	37.67
Kirare	65.28	66.51	42.06	67.00
Alebtong	22.49	22.34	20.29	26.81
Lwala	23.63	22.87	21.40	31.76
Obalanga	24.36	23.84	20.56	31.68

These values were obtained from the joint model fits to CFA and mf age-profiles data from five African LF endemic communities. The production and decay rates are, respectively, given in units of *per worm per month* and *per month,* while the survival period (*ie.*, the inverse of the decay rate) is in the unit of *month*

**Table 4:** Model-estimated CFA (at community-level as well as in 6–7 years age-cohort), and mf breakpoint values for achieving the successful interruption of LF transmission in each of the five study sites that have both CFA and mf baseline age data. As in Table 2, breakpoints are listed in terms of prevalences (%) for CFA and mf at 95 % probability of elimination for two situations: 1) at the prevailing vector biting rates (*ien*, at the observed ABRs); and 2) at the threshold biting rates (TBRs) at or below which LF transmission process cannot sustain itself regardless of the level of the infection in human hosts

Study villages	Mean TBR	95 %-EP Breakpoint values at ABRs			95 %-EP Breakpoint values at ABRs		
		CFA bpts	CFA6to7 bpts	Mf bpts	CFA bpts	CFA6to7 bpts	Mf bpts
Masaika	731	0.067185	0.004926	0.012826	0.146865	0.047919	0.136688
Kirare	497	0.089053	0.014116	0.026547	0.204496	0.056584	0.116281
Alebtong	6653	0.049452	0.002917	0.006474	0.112308	0.024612	0.05454
Lwala	3729	0.060131	0.005485	0.014214	0.12293	0.043296	0.090148
Obalanga	867	0.071218	0.010358	0.019089	0.150288	0.060039	0.127721

EP: elimination probability; bpts: breakpoints; CFA6to7: Circulating Filarial Antigen in 6 to 7 years old.

### Impact of local dynamics and interventions on LF elimination

We used the locally calibrated LF models together with their corresponding site-specific mf prevalence breakpoint values signifying a 95 % probability of elimination to simulate the impacts that various LF strategies may have on the durations (in years) required to eliminate LF from the communities under investigation. We considered the following set of intervention scenarios: 1) annual MDA (a) without and (b) with VC, and 2) biennial MDA (a) without and (b) with VC. Two main drug regimens were considered: DEC-ALB (for the study sites from PNG and in Southeast Asia), and IVM-ALB for the African sites (*cf.* Table 1). These scenarios were chosen to facilitate a comparison of the existing WHO recommended strategy of using annual MDA alone, versus the prospects that including vector control into MDA programs and switching to biennial MDA may have in potentially accelerating elimination of the disease locally. The analysis was carried out by subjecting each of the 500 SIR-resampled parameter sets estimated from a site to the drug regimen (*ie.* either DEC+ ALB or IVM + ALB) recommended for use in that setting, and assessing the number of years of intervention which would be required for all the ensemble model vectors to cross below their respective mf % breakpoint thresholds signifying a 95 % probability of LF elimination. Mf % breakpoint thresholds at ABR were used as targets when modelling the impact of MDA alone, whereas breakpoint prevalence values at TBR were used when modelling the impact of including VC, given that reducing the vector population will push the system towards the TBR breakpoint and hence raise mf breakpoints to their maximal values (Table 2). Note also that when modelling the impact of MDA alone, we assume that the prevailing ABR in a site does not change significantly over the entire period of the MDA. We have previously shown that any year-to-year change in ABR will need to be >50 % if such changes are to impact the transmission dynamics in a site [18]. We assume here that changes of this magnitude are unlikely to have occurred in our sites either due to natural or development-related causes.

**Fig. 4:**
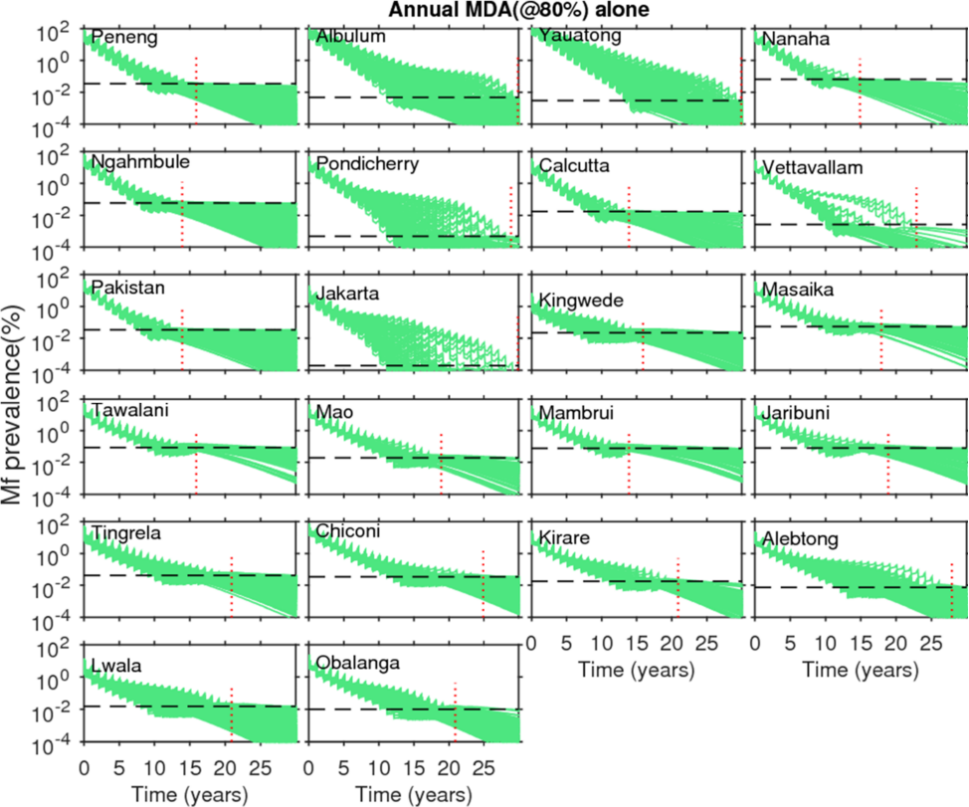
Impact of annual mass drug administration (MDA) alone at 80 % coverage on the model-predicted community-level microfilariae (mf) prevalences of lymphatic filariasis (LF) for each study site. Note that the prevalence values on the y-axis are on a *logarithmic* scale. Simulations at MDA coverage of 80 % were carried out for three decades using the best-fit parameter vectors obtained by model-fitting to the baseline mf age-prevalence data in each site (*cf.* Fig. 1). The MDA start time is indicated by 0 on the x-axis. The *horizontal* dashed line in each plot represents the model-derived extinction threshold signifying 95 % probability of elimination (site-specific numerical values are provided in Table 2), whereas the vertical dashed line denotes the time-point since the start of mass treatment at which the modelled community-level mf prevalences had reduced/crossed below their respective 95 %-EP threshold values for *all* the best parameter vectors. Note that in these simulations, models were run forward for each site without the effect of drug treatments after the time-points indicated by the vertical lines were crossed

**Fig. 5:**
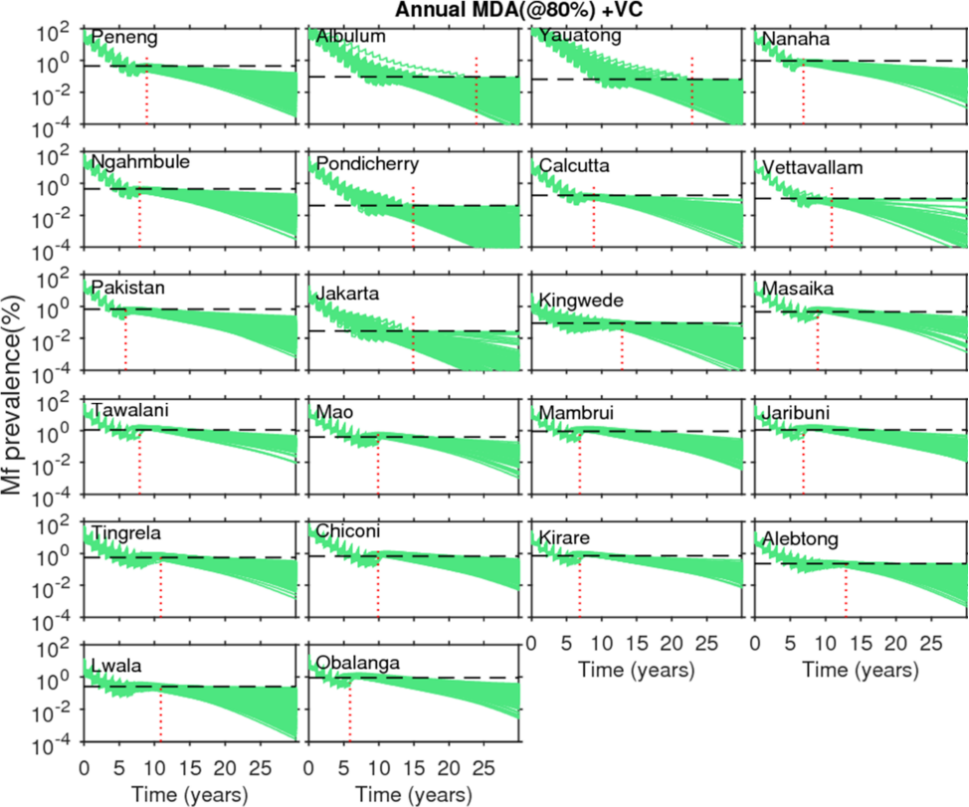
Impact of annual mass drug administration (MDA) at 80 % coverage with supplemental vector control (VC) on the model-predicted community-level microfilariae (mf) prevalence of lymphatic filariasis (LF) for each study site. The supplemental VC was implemented at the population-level coverage of 80 %, and its effect was continued throughout the model simulation period, *ie.* even beyond the MDA stopping time-point indicated by the vertical lines. All other details as described in Fig. 4

Figs. 4 and 5 show the changes in model-predicted community-level mf prevalences arising from the application of annual MDA, without (Fig. 4) and with supplemental use of VC (Fig. 5), respectively. Results are shown for coverage levels of 80 % for MDA and VC where appropriate, with drug interventions simulated for each of the SIR selected parameter vectors until the time points when predicted mf prevalences crossed below the mf breakpoint threshold values representing the 95 % probability of elimination (95 %-EP, henceforth) in each site. In the figures, the *horizontal* dashed lines represents the site-specific 95 % EP mf threshold value, while the *vertical* dotted line depicts the time point when all the SIR vectors cross below the 95 % EP mf threshold value in a site. The results depicted in Fig. 4 for annual MDA alone, indicate, firstly, that for all 22 sites this strategy even at 80 % drug coverage will require to be applied for more than a decade, and in some cases beyond 30 years, before all the parameter vectors cross the estimated 95 % EP mf value in a site. This *maximum* duration (*ie* the time point when all site-specific SIR vectors cross the 95 % EP mf value for that site) required for the stopping of MDA also varied across the 22 sites, with timelines to extinction varying strikingly from 13 to over 30 years. When VC is used as a supplement to annual MDA, the maximum duration of interventions is reduced drastically (Fig. 5). For example, for Chiconi, while using the annual MDA alone strategy required about 25 years to bring about parasite elimination (Fig. 4), this was brought down to a much more manageable 10 years when VC was included into this MDA strategy (Fig. 5). The most drastic reductions, however, were achieved by the addition of VC for those sites which exhibited initially low baseline mf prevalences compared to the sites that began with high baseline mf prevalences. Thus, for the PNG villages of Albulum (80 % mf) and Yauatong (92 % mf) which exhibited the highest baseline prevalences, among these sites, the reduction in the maximum duration by the introduction of VC was moderate (about 8 years), meaning that more than 20 years would still be required to bring about LF elimination in these sites with annual MDA even with the inclusion of VC (Fig. 5).

Table 5 gives the *mean* durations (*ie*. the average time that all SIR parameters in a site took to cross its estimated 95 % EP mf breakpoint value) of drug administrations required to achieve LF elimination in each of 22 study sites under all four strategies considered here. The results are shown for three drug coverages, *viz*., 60, 80 and 100 %, and indicate that mean durations of drug treatments required to disrupt LF transmission in each site will vary according to MDA coverage but also with the type of intervention applied. Thus, while increasing MDA coverage reduced the calculated mean years of interventions required across all sites and for all intervention scenarios (Table 5), the most significant reductions in intervention years were achieved either when MDA alone is switched from yearly to 6 monthly (*ie.* a shift from annual to biennial MDA) or when mass treatment is supplemented with VC applications, most dramatically when included in the biennial MDA strategy (the mean number of years of intervention reducing from between 10 to 23 years for the MDA alone strategy to between only 2 to 8 years for the biennial MDA plus VC strategy at 80 % drug coverages). The supplemental use of VC not only reduced the mean duration of the required years of mass treatments across all the communities considered here for both types of MDA, it also suppressed the variance in the model predictions of the mean number of years of mass treatments required to achieve LF elimination across sites (Table 5).

**Table 5:** Model-predicted required mean number of years of mass treatments for different intervention scenarios for achieving the successful interruption of LF transmission in each of the study sites investigated. The required years of mass treatments were determined by evaluating whether as a result of intervention, the model-predicted community-level mf prevalence had reduced below the mf elimination threshold signifying 95 % probability of elimination for the four scenarios: 1) intervention by annual mass drug administration (MDA) alone; 2) annual MDA with supplemental vector control (VC); 3) biennial MDA alone; and 4) biennial MDA with supplemental VC. VC, where applicable, was implemented at the community coverage of 80 %. The results in this table are shown for three MDA coverages: 60, 80 and 100 %

	Annual MDA alone			Annual MDA + VC			Biennial MDA alone			Biennial MDA + VC		
Study villages	60 %	^a^80%	100 %	60 %	^a^80%	100 %	60 %	^a^80%	100 %	60 %	^a^80%	100 %
Peneng	17	12	9	10	7	6	9	6	5	5	4	3
Albulum	26	20	15	17	12	9	15	11	10	9	7	6
Yauatong	28	23	18	19	14	11	18	14	13	10	8	7
Nanaha	15	10	8	8	6	4	8	5	5	4	3	2
Ngahmbule	15	10	8	9	7	5	8	5	4	5	3	2
Tawalani	18	13	10	9	7	5	10	7	4	5	3	1
Jaribuni	17	13	9	8	6	4	9	6	4	4	2	1
Tringela	20	14	11	10	8	6	11	7	4	5	3	1
Chiconi	22	16	13	12	9	7	12	9	5	6	4	2
Masaika	18	13	10	10	8	6	10	7	4	5	3	1
Kirare	21	16	12	8	6	4	12	8	4	4	2	1
Alebtong	25	19	14	12	9	7	15	10	6	7	4	2
Lwala	20	14	11	9	7	5	11	8	4	5	3	1
Obalanga	22	16	13	7	5	4	13	9	5	3	2	1
Kingwede	16	11	8	10	8	6	8	6	3	5	3	1
Mao	21	16	12	11	8	6	12	8	5	6	4	1
Mambrui	17	12	9	8	6	4	9	6	4	4	2	1
Pakistan	15	10	8	8	6	4	8	5	5	4	3	2
Pondicherry	24	18	13	15	11	8	13	9	8	8	6	5
Calcutta	15	11	8	9	7	5	8	5	4	5	3	2
Vettavallam	19	13	10	11	8	6	10	7	6	6	4	3
Jakarta	25	21	16	17	13	9	16	12	9	9	6	5
^b^Total variance(*An*)		3.52			2.66			1.37			1.15	
^b^Total variance(*Cx*)		1.52			1.20			0.51			0.49	

^a^The required years of mass treatments at the MDA coverage of 80 %, without and with vector control, significantly differed within *Anopheline* and *Culicine* settings (Kruskal-Wallis test *p*-values <0.0001)

^b^Total variance denotes the overall variance in the required years of mass treatments estimated from all sites within each of the Anopheline and Culicine settings. Data are provided for 80 % coverage for annual and biennial MDAs without and with vector control at 80 % coverage

### Impact of LF MDA intervention on CFA

**Fig. 6:**
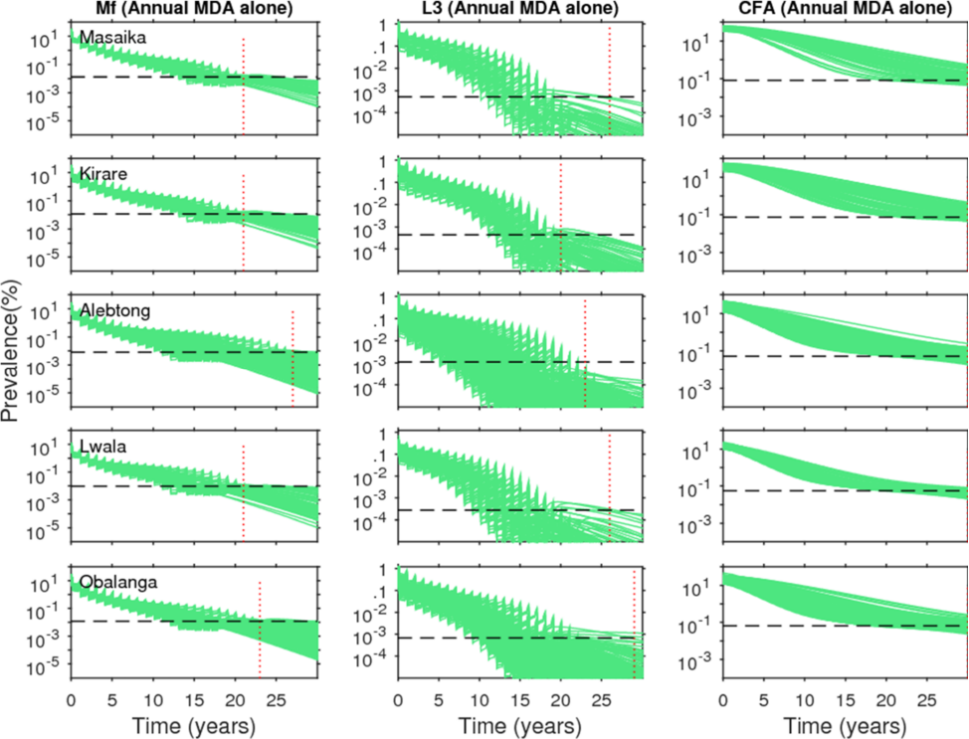
Impact of annual mass drug administration (MDA) alone at 80 % coverage on the model-predicted community-level microfilariae (mf), third-stage larvae (L3) and circulating filarial antigen (CFA) prevalences of lymphatic filariasis (LF) for the five study sites that provided both mf and CFA baseline age data. The intervention simulations were carried out with the best model parameters obtained by joint fitting to both CFA and mf baseline data. The *horizontal* dashed line in each plot represents the model-derived extinction threshold signifying 95 % probability of elimination for the respective state variables, whereas the vertical dashed line denotes the time-point since the start of mass treatment at which the modelled prevalences had reduced/crossed below their respective 95 %-EP threshold values for *all* the best parameter vectors in the case of mf and L3 prevalences; beyond this time-point model runs were carried out without the effect of drug treatments as in Figs. 4 and 5. Note that for CFA, thresholds were crossed only much after 30 years (data not shown)

The derivation of SIR parameter vectors following the joint fitting of the relevant LF model to parallel CFA and mf age-profile data available from 5 of our African study communities has allowed us to undertake a first analysis of the relative impacts of using breakpoint values from 3 LF infection indicators, *viz.* mf, L3 and CFA, as targets in interventions aiming to break LF transmission*.* The results on the impact of the annual MDA alone strategy on timelines for each of these indicators to cross their respective 95 % EP values estimated in each site are depicted in Fig. 6 (similar results were obtained qualitatively for the other 3 scenarios, and so not shown). As in Figs. 4 and 5, the horizontal lines in each plot shows the site-specific 95 % EP value estimated for each respective indicator, while the vertical lines denote the time points at which the annual MDA alone strategy caused all prevalence curves pertaining to each indicator to cross below their corresponding 95 % EP values. The major result depicted in the figure is that whereas elimination using annual MDA was shown to occur earlier and at around the same times when mf and L3 thresholds were deployed to serve as elimination targets in all the 5 study sites, none of the CFA prevalence trajectories crossed their respective site-specific 95 % EP thresholds by the end of the simulations (*ie.* by 30 years) in each of these sites (Fig. 6).

### Assessment of extinction and recrudescence probabilities from applying the WHO mf elimination threshold

**Fig. 7:**
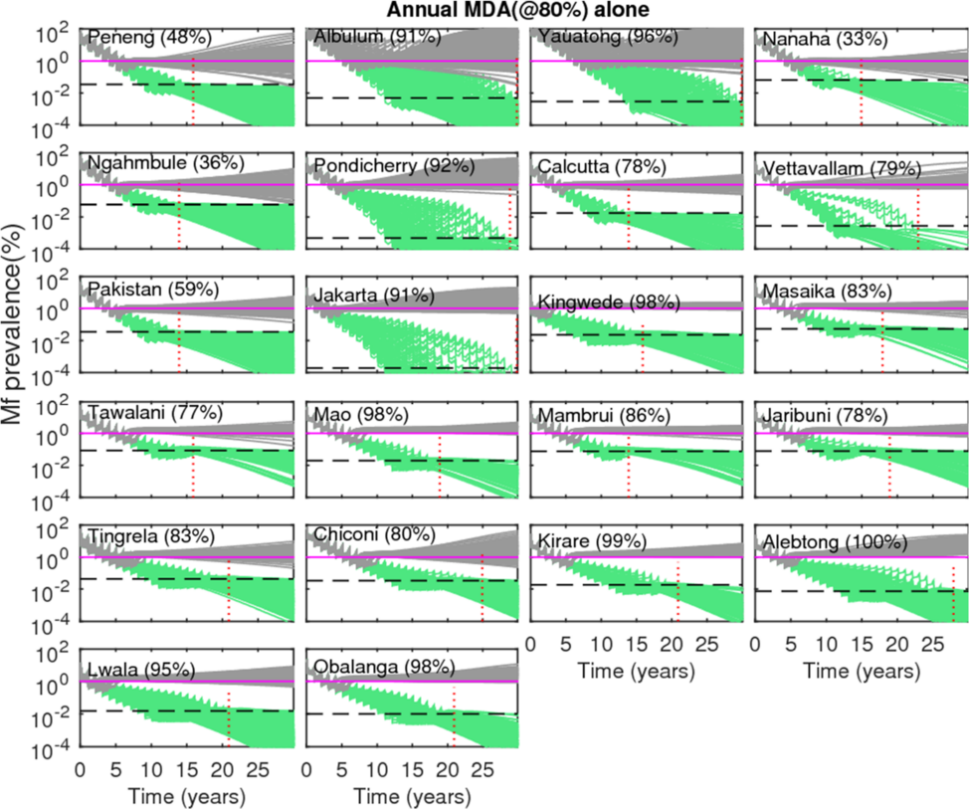
Risk of recrudescence of LF in communities as a result of the stopping of mass treatment following the WHO-recommended threshold of 1 % community-level microfilariae (mf) prevalence. Results shown in gray are for the LF intervention scenario when mass treatments (annual MDAs at 80 % coverage with no supplemental vector control) were stopped after the overall modelled mf prevalences crossed below the WHO-recommended threshold of 1 % (shown by *solid* horizontal line) in each site, whereas in the case of green curves, mass treatments were stopped after the modelled prevalences had reduced below the model-derived 95 %-EP thresholds (depicted by dashed horizontal line, for values *cf.* Table 2) in a site. Note that MDA stopping times for these thresholds varied within each site, with the vertical dotted line denoting the time-point when all model runs in a site had crossed the 95 %-EP threshold estimated for that site. Note that when modelled prevalences cross the 95 % EP in a site, all further simulated prevalences decayed steadily to the 0 state attractor, as predicted by theory [7]. The recrudescence probabilities (calculated as the percentage of the total model runs in which the mf prevalence rose above the 1 % threshold by the end of the simulation period) are provided in parentheses beside the names of the study sites

**Fig. 8:**
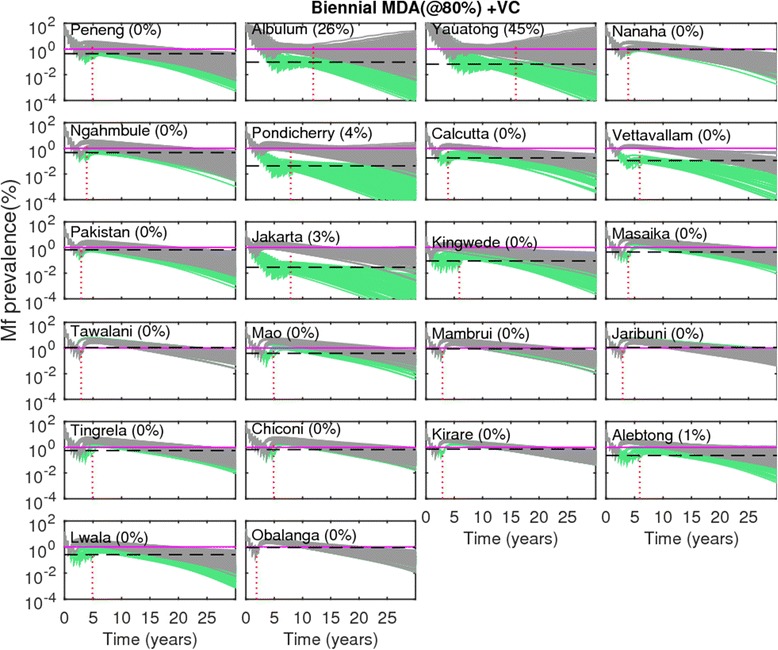
Risk of recrudescence of LF in communities as a result of the stopping of mass treatment following the WHO-recommended threshold of 1 % community-level microfilariae (mf) prevalence. These results are shown for the biennial MDA with supplemental vector control. The coverage levels in this set of intervention runs, for both MDA and VC, were kept at 80 %. All other details, including color codes, are as given in Fig. 7

Table 6 presents the probabilities of LF extinction expected in each of our 22 study sites as a result of using the WHO-recommended threshold of 1 % mf prevalence for signifying the achievement of LF transmission interruption in treated populations [50]. The results show that the probability of LF extinction using this 1 % threshold to decide if transmission has stopped in relation to the model-predicted mf breakpoint values estimated in each individual site (Table 2) was markedly low at their prevailing unperturbed ABRs, ranging from 0 % to only 31 % across these sites (Table 6). By contrast, as site-specific mf breakpoint values per site will increase to maximal values at TBR, the extinction probability using the WHO threshold of 1 % mf also increased significantly in these sites at their TBR values (range of 25 % to as high as 96 %), providing a further added impetus for the need to consider including vector control in MDA programs, *viz.* that such a strategy will also yield the possibility of using higher and more easily measurable breakpoints to determine if parasite elimination has occurred. The implications of using the WHO threshold for risk of LF recrudescence after MDA is stopped following the crossing of the simulated community-level mf prevalences below the 1 % WHO target in each study are displayed in Figs. 7 and 8. Figure 7 depicts the resulting trajectories of the model-predicted community-level mf prevalences for the annual MDA alone intervention strategy, while Fig. 8 portrays the results for the most effective strategy studied here, *viz.* the biennial MDA supplemented with VC strategy. The simulations show that the risk of recrudescence can be quite high, with probability values ranging from 33 to 100 %, in the case of the annual MDA alone strategy (Fig. 7). By contrast, for the intervention strategy of biennial MDA supplemented with VC, the risk of recrudescence was found to be significantly much lower (<5 %) to zero for the majority of the sites, with only 2 highly endemic PNG sites (*eg.*, Albulum and Yauatong) showing a substantial risk of recrudescence (Fig. 8).

**Table 6:** Probability of LF extinction at the WHO threshold of 1 % mf prevalence. See text for details of estimation

Study villages	Probability of extinction at ABR	Probability of extinction at TBR
Peneng	7.75	89.59
Albulum	0.43	40.86
Yauatong	0.54	36.93
Nanaha	16.67	95.24
Ngahmbule	14.55	92.99
Tawalani	16.3	96.3
Jaribuni	21.79	96.15
Tingrela	15.38	79.49
Chiconi	7.94	93.33
Masaika	23.6	89.89
Kirare	16.71	36.86
Alebtong	0.95	71.43
Lwala	0	74.49
Obalanga	0	78.89
Kingwede	3.67	48.93
Mao	4.44	90
Mambrui	31.31	95.96
Pondicherry	6.9	34.48
Calcutta	2.73	66.36
Vettavallam	0	55.77
Pakistan	9.45	93.44
Jakarta	18.98	24.82

## Discussion

We have introduced a data-model assimilation methodology founded on the Bayesian calibration of mechanistic simulation models to address the urgent need for developing robust modelling tools that can effectively support the management of parasitic disease elimination programs. The developed framework is based on a parameter estimation and calibration technique called Bayesian Melding (BM) which aims to combine the advantageous features of both mechanistic and statistical approaches to yield models that are based on mechanistic understanding yet remain with the bounds of data-based parameter estimation [10, 18, 28, 29, 35, 45, 51–55]. Such data-model assimilation approaches have been shown to significantly improve making inferences from simulation models, and in their predictions, for a wide range of modelling applications in natural systems, including in ecology, climatology, fisheries and increasingly infectious diseases [31, 32, 56]. In particular, this body of work has shown how these approaches, by using data to constrain a model during simulation to yield results that approximate reality as closely as possible, can allow better inference or predictions of the most likely future state of a natural system, while at the same time also adequately facilitating the capture of the impact that local variations might have on system dynamics [10, 18, 26, 27, 57, 58]. This is a more stringent set of requirements than applied to models for producing plausible futures consistent with theoretical understanding alone, which are also generally based on the assumption of constancy in rate parameters over time and space. If biological understanding, assumptions and parameter values of such averaged models are incorrect, then the resulting predictions may be inadequate to capture the dynamics of an ecological system, especially in the local context. Furthermore, besides limited epistemic understanding, when ecological complexity is high and data are limited, as is often the case with parasitic infection and intervention data, efficient data-model integration to support inference and predictions is crucial [31].

Here, our contribution is to illustrate how a BM based data-model assimilation framework may offer a platform for reliably integrating LF transmission models with diverse infection data, including site-specific information on the key drivers of parasite transmission, for the purpose of: 1) improving learning about the transmission process, and 2) aiding the assessment of the effectiveness of alternative disease elimination management strategies for breaking transmission with high levels of confidence across disparate community settings. BM is a versatile framework for coupling dynamic models with data that has the chief feature that it retains the desirable properties of standard Bayesian inference for permitting the drawing of robust conclusions from deterministic models via effective synthesis of information from models and data, but modifies the process so as to avoid the Borel paradox that can affect such synthesis, *ie.* the situation where postmodel distributions are dependent on or biased by how the simulation model is parameterized [35, 59]. The BM approach we have developed is also efficient because model calibrations can be carried out reasonably fast mainly because we use the relatively simple SIR algorithm to simulate from the posterior distribution [35, 60]. While Markov Chain Monte Carlo (MCMC) methods, *eg.* the Gibbs sampler [61], or more generally, the Metropolis-Hastings algorithm [62], have been developed for this purpose in recent years, computing the analytic posterior density is known to be cumbersome for such approaches. Besides this, the posterior parameter distributions for complex systems, such as the LF system, are invariably also likely to exhibit strong dependencies [26, 27]; these are again difficult to resolve using common MCMC algorithms [29]. For these reasons, and given that we are able to simultaneously accomplish the various goals we set out to undertake using the BM formulation we have developed, *ie.* offer insights into the degree of information that typical survey data contain about model inputs and parameters, and the obtaining of satisfactory predictions along with uncertainty bounds for modelled output variables of chief interest, we believe the approach described here works well and is simpler and efficient to apply.

Our first major result from this study based on the BM calibration of LF models to the most extensive parasitological and entomological field data assembled to date on community infection patterns, is that, as a result of effective parameter constraining by local transmission conditions (see model fits to data and parameter calibration results given in Figs. 1 and 2), significant variations in population dynamics and in the resultant transmission and infection breakpoints occurred between the 22 LF endemic communities investigated (Table 2). This pattern was observed irrespective of the LF endemic region or vector species implicated in the transmission of the parasite (Tables 1 and 2), and re-emphasizes our previous conclusion, based on the modelling of a smaller subset of these data [10], regarding the important need for effectively assimilating locality-specific infection data into process-based mathematical models as a strategic framework for determining and analysing LF elimination endpoints. Further, the demonstration that the values of thresholds are ultimately linked to the dynamical processes that cause a parasite system to shift to alternate stable states, either the extinct or the endemic state, also underscores how an understanding of parasite transmission and extinction dynamics, rather than merely using empirical field evaluations, will be vital to any attempt to define breakpoints, regardless of the transmission or infection indicator, for assessing LF elimination [7, 8, 19, 22, 46, 63–65].

Inspection of Table 2 further clarifies how given that a transmission model, by being able to couple and address all the relevant state variables together and thus is able to provide predictions of simultaneous changes in such interlinked variables, can in addition provide information on the values of the many different thresholds that could be used to assess parasite elimination for a particular transmission system [6, 8, 18, 22]. As we have shown previously [8, 22, 66], such thresholds in LF, reflecting the general dynamics of the vector-borne transmission process, can include both the threshold vector biting rate as well as thresholds arising from infection in the human as well as vector host populations [1, 6–8, 10, 18, 67]. Further, it is clear that the latter infection thresholds can also be quantified by a transmission model for any specific diagnostic tool that can be used to estimate infection prevalence or levels, *eg.* in the present case whether by microscopy or PCR-based techniques to detect mf in human blood samples or L3 larvae in mosquitoes, or via the use of immunological tests to measure circulating filarial antigens (CFA). The values in Tables 2 and 4 portray another important dynamical feature of infection breakpoint values, which is not fully appreciated by disease control managers, and that is that the maximal value of these variables is attained at the transmission threshold (TBR) and will be significantly smaller than this maximal value (itself typically small) at the prevailing vector biting rate ABR, particularly if such ABRs in a site are large. Note this result immediately indicates how adding vector control to mass treatments, by increasing infection breakpoints as ABR is reduced, can enhance the prospects of LF transmission interruption.

The Monte-Carlo-based sampling technique used in our BM procedure for model fitting as well as for deriving results by taking samples from the postmodel distribution has elucidated how, rather than being a single estimate, both infection-related and vector biting abundance thresholds in LF may exist as a range of values within and between village sites. This supports the notion that uncertainty and stochastic variability in transmission parameters will invariably give rise to a distribution of these variables in natural communities [10, 68–70], an outcome which clearly has significant strategic implications for elimination programs, as it implies that any one value from such distributions in a site may be chosen to serve as an infection breakpoint target. However, as each value from the ensemble will be associated with a probability of elimination if crossed, this finding also clarifies that the choice of which of these values to use is ultimately driven by how risk of program failure is perceived and accepted by the relevant policy makers, *ie.* whether management or the decision maker is risk averse (and hence opts for high confidence (*eg.* 95 % probability) of achieving elimination) or risk tolerant (and so is tolerant of using values signifying lower confidences of achieving elimination). Typically, choosing higher values from such a distribution will provide lower probabilities of elimination, while choosing and using lower values from the same distribution will give higher probabilities of elimination [11, 70]. Here, thus, while we focus on the use of infection values reflecting a 95 % probability of elimination as breakpoint targets, it is important to bear this trade-off in mind when interpreting the conclusions presented in the following.

The values of breakpoints tabulated in Tables 2 and 4 are the first estimates produced for all the various transmission and infection variables that could serve as extinction targets for LF [8, 19, 22, 23]. Perusal of the results indicates that a key feature of these estimates is that apart from exhibiting significant site-to-site heterogeneity, their actual numerical values are typically very small, even in the case of TBR values when compared to the prevailing ABR values of each site. By comparison, the WHO appears to recommend the use of reducing mf to below 1 % and CFA to below 2 % in the overall population in all sites following interventions and post-surveillance [50], as criteria for determining if sustained LF has occurred in a region. If the breakpoint values we have estimated in this paper are correct- and we have previously produced the first empirical evidence that these model-estimated thresholds could indeed occur around at the levels reported here in endemic communities [47] - then clearly applying the WHO thresholds would severely overestimate the possibility that transmission has been achieved in each of our sites. This likelihood is illustrated by the results presented in Table 6, which give the probability that LF elimination has been met in each of our study site by applying the WHO criterion of reducing mf prevalence to <1 %, given the distribution of mf breakpoint prevalence values estimated in each of these sites. The results illustrate that if the model-estimated mf breakpoint values hold, then using the globally set WHO threshold of 1 % will have significantly overestimated the attainment of LF transmission cessation in the majority of these sites, with the most dramatic impact on the resultant elimination probabilities occurring where the WHO criterion is used uncritically in cases where the prevailing vector populations are not disturbed, *ie.* when MDA is used alone (the use of the 1 % mf prevalence threshold yielding between 0 % to at best only 31.31 % probabilities of elimination). This is because mf breakpoint values at the prevailing ABR represent the targets for assessing elimination using MDA interventions, and these are considerably lower in numerical values compared to those obtaining at TBR (Table 6). While the situation of using the WHO criterion is markedly improved when compared to model-estimated mf breakpoint values at TBR, the occurrence of between-site heterogeneity in breakpoint values means that in some settings this could still give rise to a low (<50 %) probability of elimination. These results further underline a significant but very little appreciated impact of including vector control into MDA programs, *viz.* that as ABR is reduced towards TBR as a result of vector control, breakpoint values will be raised to their maximal values and thus the use of a judiciously chosen higher breakpoint target (closer to the WHO target of 1 %) could be used for assessing LF transmission cessation under such circumstances (Table 6).

Recently, considerable empirical work has been expended in investigating the use of CFA as a marker of infection, and as an indicator for determining transmission interruption in communities undergoing MDAs [63–65]. This has led to various suggestions regarding the threshold prevalence of CFA that could be used for signifying a break in LF transmission, ranging from a value of <1 to 2 % in the overall population [19, 50, 65] to <0.1 % CFA prevalence in children between the ages of 6 and 7. The implicit rationale behind these arguments is that these comparatively higher target values would be easier to achieve and quantify than using a mf-based threshold, largely owing to the widely expected higher sensitivity of the antigen test especially in the case when population infection levels are reduced to very low levels by MDA programs. Here, we have used the availability of parallel age-dependent CFA and mf prevalence data from five individual published studies to investigate the value of using CFA in LF elimination assessments based on quantifying the dynamics of change in this indicator as a function of variations in the underlying worm population [8]. Tables 3 and 4 show the results obtained from a joint fit of the relevant vector-specific LF model to the parallel CFA and mf age-prevalence data (model fits to observations given in Fig. 3) from each of the five published studies. The parameter estimates for the observed CFA dynamics in each site are given in Table 3; these provide information firstly on a key ongoing controversy in the use of antigen tests as markers of live parasitic infection, *viz.* the rates of production and decay of the antigen used for measuring infection. These show in particular that the monthly decay rates for LF CFA are likely to be low, yielding typical persistence times of between 22.5 to up to as high as 65 months in the different study communities. Although based on only 5 studies, these estimates imply not only can CFA persist for significant periods of time but also, as for other transmission parameters, that these periods may vary substantially between sites, presumably reflecting variations in the underlying worm burdens but also possibly the effects of other types of heterogeneities that are likely to affect antigen clearance, *eg.* host immunity and/or other host physiological factors related to parasite transmission [71]. Besides persistence, a second major feature - highlighted by the estimates listed in Table 4 - is that CFA breakpoint values can, as in the case of mf, also be significantly lower (orders of magnitude of between 1 and 2) than the empirical values recommended by WHO as CFA breakpoint targets for current elimination programs. As for using mf thresholds, this result again suggests the possibility that using the WHO CFA threshold values would overestimate the achievement of parasite elimination in each of the present sites. One last impact of CFA dynamics, particularly the influence of decay rates, on the utility of using CFA for assessing LF elimination, is portrayed in Fig. 6, which depicts and compares the timelines to parasite elimination using annual MDA based on the use of mf, L3 and CFA threshold values. The results show that while the use of mf and L3 prevalence thresholds would result in meeting transmission interruptions earlier and for around the same durations of MDA (between 20 to 25 years generally), CFA prevalence curves will decline much more slowly than either, reaching their estimated elimination thresholds only much later (>30 years). This indicates that there will be a lag in the intervention response of CFA, with antigen circulating in the host population long after actual transmission has been broken leading to the possibility of making severe underestimations of the effectiveness of an intervention for breaking LF transmission and hence decisions to continue with unwarranted treatments. These results highlight the vital need for considering the inherent dynamics of an infection indicator in order to establish and test values that would signify parasite transmission interruption in a community reliably.

The intervention simulations carried out in this study primarily focussed on gaining insights into two key questions regarding the impact of current or proposed interventions for eliminating LF transmission, *viz.* 1) what is the likely impact of site-specific heterogeneity in transmission dynamics, including variable community breakpoints, on the prospects of interrupting parasite transmission using the WHO-recommended drug treatment strategies alone, and with the inclusion of supplemental vector control, in different settings and 2) what the implications would be for the long-term dynamics of LF, particularly for infection recrudescence, if interventions are stopped following the achievement of the WHO-recommended threshold of 1 % community-level mf prevalence. With regard to the first question, our analysis shows unequivocally that as a result of strong, variable, localized transmission dynamics, the durations of interventions required to cross the locality-specific elimination breakpoints predicted in this work varied significantly across each of our study sites (Figs. 4 and 5; Table 5). These durations were also generally the longest and much more variable when using the annual MDA alone strategy (between 10 to 23 years at 80 % coverage, for example (Table 5)), compared to the biennial MDA and the MDA plus vector control strategies. Interestingly, the results show that overall an annual MDA plus vector control intervention and a biennial MDA alone strategy are likely to produce a similar outcome, with the simulations showing that between 5 and 14 years of interventions will be enough to achieve the interruption of transmission in the present sites, irrespective of which of these strategies is deployed (Table 5). This is a major new finding, and indicates that in settings where vector control will be difficult to implement, it might be worthwhile seriously considering moving from annual to biennial MDA if LF elimination is to be accomplished within a reasonable timeframe. The most effective strategy, however, clearly is where biennial MDA can be coupled with vector control, with the results showing that it will markedly reduce the durations of control required to achieve elimination (to between 2 to 8 years only at 80 % MDA coverage). The comparison of these results with that of the annual MDA alone strategy shows that such an intervention may well be a necessity particularly in those anopheline settings that exhibit the highest endemicity rates at baseline (Tables 1 and 5); this also implies that if a set or fixed timeframe, *eg.* the WHO target of meeting LF elimination by 2020, for achieving parasite elimination is to be followed, then a flexible strategy that adapts specific interventions to local transmission patterns in different geographic settings may well be prove to be the most cost-effective method for achieving LF elimination in a region.

The results given in Table 5 highlight a further significant strategic impact of adding vector control to MDA programs, *viz.* that incorporating such combination control will also allow the overcoming of the negative impact of failing to meet a high MDA coverage (>80 % at least) in a community, with inclusion of vector control at lower MDA coverages in our sites delivering similar durations of interventions needed to achieve elimination as that predicted for using MDA at much higher coverages (compare mean durations of control predicted for MDA alone strategies at 80 or 100 % coverages versus the durations predicted to achieve parasite elimination with inclusion of vector control at the lower 60 % MDA coverage given in Table 5). A final intriguing feature of the intervention modelling results summarized in Table 5 with respect to inclusion of vector control is that the overall variability in the number of years of interventions required to achieve LF elimination is also reduced for either the annual or biennial MDA strategies when vector control is included into these strategies. This occurred irrespective of the vector species implicated in LF transmission in the study sites, and highlight that including vector control into MDA programs may yield a countermeasure that could significantly be also robust to differential locality-specific control dynamics.

With respect to the question concerning the potential effects on long-term LF transmission dynamics and the risk of recrudescence as a result of using the WHO mf threshold versus the model-estimated mf breakpoints in a site, our comparison of mf trajectories following the crossing of these respective thresholds – portrayed in Fig. 7 for the annual MDA alone and Fig. 8 for the biennial MDA plus vector control strategy – has yielded two key insights on this important topic. First, our simulation results demonstrate that when the WHO threshold of 1 % mf prevalence is used as a threshold with an annual MDA alone strategy, there were lower probabilities of achieving eventual transmission interruption in the study sites (Table 6), and consequently high probabilities that recrudescence will occur (between 33 to 100 %) in all these sites once treatment is stopped following the crossing of this threshold (Fig. 7). By contrast, even though it will take considerably longer to cross, the use of the model estimated site-specific mf breakpoints as targets for MDA will lead to a steady decline in mf levels to the 0 state once these are crossed in each site. A second feature highlighted by the predictions displayed in Fig. 8, however, indicates that it may nonetheless be possible to derive and set a global threshold value that will prevent recrudescence in most settings; but this will depend intimately on the type of intervention pursued. Thus, the simulations shown in this figure for the biennial MDA plus vector control intervention indicate that in this case, even a high globally set threshold of 1 % mf prevalence will be sufficient to ensure permanent LF elimination, with all trajectories of infection declining, as in the case of mf curves crossing their site-specific breakpoints, towards the extinct state. This is a significant, not previously fully understood, finding and suggests how including vector control has the potential to also help define an effective higher elimination threshold that could also be possibly applicable or settable globally.

## Conclusions

In this paper, we have outlined a Bayesian data-model assimilation framework to address the need for robust methodological tools that can, by enabling rigorous assessments of the predictive uncertainty of parasite transmission models, enhance their use for supporting reliable management decision making. The proposed framework aims to combine the advantageous features of both mechanistic and statistical approaches, whereby the mechanistic basis enhances the confidence in predictions made for a variety of conditions and transmission settings, while the statistical methods provide a sound foundation for parameter estimation [28, 29, 35, 57, 58]. We have shown how this framework can refine our knowledge of model parameters from data, and obtain predictions of infection dynamics along with credible intervals for modelled output variables. The application of this approach to parallel LF infection and vector data assembled from 22 endemic sites from each of the major LF endemic regions has highlighted how major open questions connected with elimination of this parasitic disease, *viz.* estimation of elimination thresholds and quantification of the dynamics of parasite population responses to different interventions, can be resolved to provide information regarding site-specific transmission and endpoint complexities, as well as insights into the best approaches for breaking self-sustaining parasite transmission accommodating these local heterogeneities.

While these first results have enhanced our confidence in the utility of this modelling approach as a quantitative tool both for improving learning about LF transmission dynamics as well as for supporting the derivation of intervention programs adapted to local conditions, it is clear that a challenge is how to extend the developed framework for providing predictions in those settings lacking or containing only sparse data to inform the modelling process [31, 56, 72]. We suggest that there might be several ways to address this problem. A first approach, as highlighted in this study, is to uncover a strategy that is robust to between-site variability in parasite system breakpoints and intervention dynamics. We have shown in this regard how including vector control to MDA may allow the selection and use of a higher breakpoint that might also work relatively well globally across all settings as a target for signifying LF transmission interruption. Note that inclusion of vector control will also dramatically reduce the number of years of interventions required to break transmission while also reducing variability in such durations between sites, suggesting that it might also be possible to set a lower maximal duration of control, *eg.* up to 8 years when combined with biennial MDA, that would work well across all endemic settings for bringing about self-sustaining transmission interruption everywhere. Such an approach, however, is likely to only partially resolve the challenge of dealing with heterogeneous transmission and extinction dynamics, given the difficulties of achieving a suitably and sustainably high vector control coverage in many social settings, and the ever present potential for rapid behavioural and adaptive change by mosquito populations in response to chemical control [48, 73]. A second approach might be to use ensemble methods to first explore the type of model predictions that can be made for sets of possible parameter values and inputs, such as ABR or infection prevalence [74–76], followed by clustering the different predictions based on distinct combinations of parameter sets and inputs. This would allow selection of models based on variation in input variables across a region, but will still require spatial information on such variables. These difficulties suggest that ultimately we will require to develop an approach that would be able to reliably facilitate the accommodation of spatial and temporal variation in model parameter as well as input values directly. We are currently developing such a computational framework that will aim to couple the BM-based LF models to mapping systems which, by allowing spatial interpolations of ABR and mf prevalence variables across a region to serve as inputs into the BM process, would facilitate the derivation of LF models suitably parameterized to reflect the transmission conditions for any given endemic site in a region [57, 58]. Although likely to be computationally intensive, we suggest that such an approach would provide the most comprehensive solution to addressing the problem of heterogeneity in parasite population responses to interventions applied in different settings, and thus for supporting the prediction and analysis of the best approaches for achieving LF elimination everywhere. For both approaches, however, validation with post-MDA monitoring data will be key; we echo in this regard increasing calls for the assembly and release of these data to modellers from the many countries that are currently collecting these data as part of their monitoring and evaluation activities. It will also call for a more efficient approach to importance sampling to reduce computational time, for example by deploying adaptive sampling importance resampling methods [51], in which SIR is run a first time, and then the sampling function is replaced by a more efficient one (*eg.* sequential or annealed importance sampling [77]) based on first results. Finally, it will require a closer assessment of model structures and evaluation of error and bias in predictions generated by fitting to only type of commonly available data, *eg.* mf prevalence data alone, when simultaneous fitting to joint infection data, such as to parallel CFA and mf data, might allow a better constraining of model parameters and thus to significant reductions in prediction error. Current work is underway to address each of these problems.

## Additional file

Additional file 1: Table S1. Description the basic LF model parameters and functions used in the model. (PDF 63 kb)

## Abbreviations

ABR: Annual biting rate
ALB: Albendazole
BM: Bayesian melding
Bpts: Breakpoints
CFA: Circulating Filarial Antigen
DEC: Diethylcarbamazine citrate
EP: Elimination probability
IVM: Ivermectin
LF: Lymphatic filariasis
LLINs: Long lasting insecticidal nets
MBR: Monthly biting rate
MDA: Mass drug administration
mf: Microfilariae
SIR: Sample importance resampling
TBR: Threshold biting rate
VC: Vector control
WHO: World Health Organization
