# Supplementary material for: Bayesian calibration of simulation models for supporting management of the elimination of the macroparasitic disease, Lymphatic Filariasis
Source: Parasit Vectors. 2015 Oct 22;8:522. doi: 10.1186/s13071-015-1132-7 (PMC4618871; doi:10.1186/s13071-015-1132-7)
Supplement: Additional file 1: Table S1. — Description the basic LF model parameters and functions used in the model. (PDF 63 kb) [file 13071_2015_1132_MOESM1_ESM.pdf]

Additional file 1

Table S1 - Description the basic LF model parameters and functions used in the model.

Parameter Symbol	Definition (*units*)	Range	Source
Intrinsic ***Biological*** parameters			
λ	Number of bites per mosquito (*per month*)	[5, 15]	[1]-[5]
τ	Pre-patency period (*months*)	[6, 9]	[6]
s	Proportion of female worms	0.5	-
μ	Worm mortality rate (*per month*)	[0.008, 0.018]	[3]-[5],[7]-[10]
α	Production rate of microfilariae per worm (*per month*)	[0.25, 1.5]	[3]-[5],[11]
γ	Death rate of the microfilariae (*per month*)	[0.08, 0.12]	[4],[5],[9],[11]
α_2	Production rate of CFA (per *worm per month*)	[2, 8]	This study
γ_2	Decay rate of CFA (*per month*)	[0.01, 0.05]	This study
g	Proportion of mosquitoes which pick up infection when biting an infected host	[0.259, 0.481]	[4],[5],[12]
κ	Maximum level of L3 given mf density	[3.955, 4.83]	[4],[5]
k_0	The basic location parameter of negative binomial distribution used in aggregation parameter ($k = k_0 + k_{Lin}M$)	[0.000036, 0.00077]	[4],[5],[13],[14]
δ	Immunity waning rate (*per month*)	[0, 0.000001]	[4],[5]
Extrinsic ***Biological*** parameters			
V/H	Ratio of number of vector to hosts	MBR / λ	Data (Table 1)
k_{Lin}	The linear rate of increase in the aggregation parameter defined above	[0.00000024, 0.282]	[4],[5],[13],[14]
σ	Death rate of mosquitoes (*per month*)	[1.5, 8.5]	[4],[5],[15]
ψ_1	Proportion of L3 leaving mosquito per bite	[0.1, 0.8]	[11]
ψ_2	The establishment rate[1]	[0.0000398, 0.00364]	[3]-[5],[15]
#H_{Lin}	A threshold value used in $h(a)$ to adjust the rate at which individuals of age a are bitten: linear rise from 0 at age zero to 1 at age H_{lin} in years. $h(a) = a / H_{Lin}$ for $a < H_{Lin}; h(a) = 1$ for $a \geq H_{Lin}$	[20, 50]	Data (Table 1)
r	Gradient of mf uptake[2]	[0.04, 0.25]	[4],[5]
c	Strength of acquired immunity	[0.0000003, 0.0109]	[4],[5]
I_C	Strength of immunosuppression[3]	[0.5, 5.5]	[4],[5]
S_C	Slope of immunosuppression function[4] (*per worm/month*)	[0.01, 0.20]	[4],[5]
Description of the functions used in the model			
**Function**	**Mathematical expression**	**Parameters**	**Source**
#Probability that an individual is of age *a*	$\pi(a) = A_0 \exp[-B_0 a]$	a - human age	[4],[5],[16]
Adult worm mating	$\phi[W(a,t),k] = 1 - \left(1 + \frac{W(a,t)}{2k}\right)^{-(1+k)}$	k - negative binomial aggregation parameter	[3]-[5],[17]

probability			
Immunity to larval establishment	$g_1[I(a,t)] = \dfrac{1}{1 + cI(a,t)}$	c - strength of immunity to larval establishment	[4],[5]
Host immune-suppression	$g_2[W_T(a,t)] = \dfrac{1 + I_C S_C W_T(a,t)}{1 + S_C W_T(a,t)}$	I_C - strength of immunosuppression; S_C - slope of immunosuppression	[4],[5]

[1]The proportion of L3-stage larvae infecting human hosts that survive to develop into adult worms [4].
[2]The gradient of mf uptake r is a measure of the initial increase in the infective L3 larvae uptake by vector as M increases from 0 [4],[16].
[3] The facilitated establishment rate of adult worms due to parasite-induced immunosuppression in a heavily infected human host
[4] The initial rate of increase by which the strength of immunosuppression is achieved as W increases from 0 [18].
[#]The parameters A_0 and B_0 are estimated from the human demographic data of a country. Similarly, the range of H_{Lin} were derived from the LF baseline mf/CFA age data.

The term $f[M(a,t)]$ describes the functional form relating the L3-stage larval uptake and development in the vector population.

For *Anopheles* mosquitoes:

$$f[M(a,t)] = \left[\frac{2}{\left[1 + \frac{M(a,t)}{k}\left(1 - \exp\left[-\frac{r}{\kappa}\right]\right)\right]^k} - \frac{1}{\left[1 + \frac{M(a,t)}{k}\left(1 - \exp\left[-\frac{2r}{\kappa}\right]\right)\right]^k} \right].$$

For *Culex* mosquitoes:

$$f[M(a,t)] = \left(1 + \frac{M(a,t)}{k}\left(1 - \exp\left[-\frac{r}{\kappa}\right]\right)\right)^{-k}.$$

References

1. Rajagopalan PK. Population dynamics of Culex pipiens fatigans, the filariasis vector, in Pondicherry - influence of climate and environment. Proc Ind Nat Science Acad. 1980;B46:745-752.

2. Subramanian S, Manoharan A, Ramaiah KD, Das PK. Rates of Acquisition and Loss of Wuchereria Bancrofti Infection in Culex Quinquefasciatus. Am J Trop Med Hyg. 1994;51 2:244-249.

3. Gambhir M, Michael E. Complex ecological dynamics and eradicability of the vector borne macroparasitic disease, lymphatic filariasis. PLoS One. 2008;3 8:e2874; doi:10.1371/journal.pone.0002874.

4. Gambhir M, Bockarie M, Tisch D, Kazura J, Remais J, Spear R, et al. Geographic and ecologic heterogeneity in elimination thresholds for the major vector-borne helminthic disease, lymphatic filariasis. BMC Biol. 2010;8:22; doi:10.1186/1741-7007-8-22.

5. Singh BK, Bockarie MJ, Gambhir M, Siba PM, Tisch DJ, Kazura J, et al. Sequential modeling of the effects of mass drug treatments on Anopheline-mediated lymphatic filariasis infection in Papua New Guinea. PLoS One. 2013;8 6:e67004; doi:10.1371/journal.pone.0067004.

6. Scott AL. Lymphatic-dwelling Filariae. In: Nutman TB, editor. Lymphatic Filariasis. London: Imperial College Press; 2000. p. 5-39.

7. Vanamail P, Subramanian S, Das PK, Pani SP, Rajagopalan PK. Estimation of fecundic life span of Wuchereria bancrofti from longitudinal study of human infection in an endemic area of Pondicherry (south India). Indian J Med Res. 1990;91 July:293-297.

8. Evans DB, Gelband H, Vlassoff C. Social and economic factors and the control of lymphatic filariasis: A review. Acta Trop. 1993;53 1:1-26; doi:10.1016/0001-706X(93)90002-S.

9. Ottesen EA, Ramachandran CP. Lymphatic Filariasis Infection and Disease - Control Strategies. Parasitol Today. 1995;11 4:129-131.

10. Vanamail P, Ramaiah KD, Pani SP, Das PK, Grenfell BT, Bundy DAP. Estimation of the fecund life span of Wuchereria bancrofti in an endemic area. Trans R Soc Trop Med Hyg. 1996;90 2:119-121.

11. Hairston NG, de Meillon B. On the inefficiency of transmission of Wuchereria bancrofti from mosquito to human host. Bull World Health Organ. 1968;38 6:935-941.

12. Subramanian S, Krishnamoorthy K, Ramaiah KD, Habbema JDF, Das PK, Plaisier AP. The relationship between microfilarial load in the human host and uptake and development of Wuchereria bancrofti microfilariae by Culex quinquefasciatus: a study under natural conditions. Parasitology. 1998;116 03:243-255.

13. Subramanian S, Pani SP, Das PK, Rajagopalan PK. Bancroftian Filariasis in Pondicherry, South India: 2. Epidemiological Evaluation of the Effect of Vector Control. Epidemiol Infect. 1989;103 3:693-702.

14. Das PK, Manoharan A, Subramanian S, Ramaiah KD, Pani SP, Rajavel AR, et al. Bancroftian Filariasis in Pondicherry, South India: Epidemiological Impact of Recovery of the Vector Population. Epidemiol Infect. 1992;108 3:483-493.

15. Ho BC, Ewert A. Experimental transmission of filarial larvae in relation to feeding behaviour of the mosquito vectors. Trans R Soc Trop Med Hyg. 1967;61 5:663-666; doi:10.1016/0035-9203(67)90130-7.

16. Norman RA, Chan MS, Srividya A, Pani SP, Ramaiah KD, Vanamail P, et al. EPIFIL: the development of an age-structured model for describing the transmission dynamics and control of lymphatic filariasis. Epidemiol Infect. 2000;124 3:529-41.

17. May RM. Togetherness among Schistosomes: its effects on the dynamics of the infection. Math Biosci. 1977;35 3–4:301-343; doi:10.1016/0025-5564(77)90030-X.

18. Duerr HP, Dietz K, Eichner M. Determinants of the eradicability of filarial infections: a conceptual approach. Trends Parasitol. 2005;21 2:88-96; doi:10.1016/j.pt.2004.11.011.
